# Disrupted control of origin activation compromises genome integrity upon destabilization of Polε and dysfunction of the TRP53-CDKN1A/P21 axis

**DOI:** 10.1016/j.celrep.2022.110871

**Published:** 2022-05-31

**Authors:** Valerie Borel, Stefan Boeing, Niek Van Wietmarschen, Sriram Sridharan, Bethany Rebekah Hill, Luigi Ombrato, Jimena Perez-Lloret, Deb Jackson, Robert Goldstone, Simon J. Boulton, Andre Nussenzweig, Roberto Bellelli

**Affiliations:** 1 The Francis Crick Institute, 1 Midland Road, NW1 1AT London, UK; 2 Laboratory of Genome Integrity, National Cancer Institute, NIH, Bethesda, MD, USA; 3 Centre for Cancer Cell and Molecular Biology, The Barts Cancer Institute, Queen Mary University of London, Charterhouse Square, Barbican, EC1M 6BE London, UK; 4 Centre for Tumour Microenvironment, The Barts Cancer Institute, Queen Mary University of London, Charterhouse Square, Barbican, EC1M 6BE London, UK

**Keywords:** Polε, TRP53, CDKN1A/P21, DNA replication, genome stability

## Abstract

The maintenance of genome stability relies on coordinated control of origin activation and replication fork progression. How the interplay between these processes influences human genetic disease and cancer remains incompletely characterized. Here we show that mouse cells featuring Polε instability exhibit impaired genome-wide activation of DNA replication origins, in an origin-location-independent manner. Strikingly, *Trp53* ablation in primary Polε hypomorphic cells increased Polε levels and origin activation and reduced DNA damage in a transcription-dependent manner. Transcriptome analysis of primary *Trp53* knockout cells revealed that the TRP53-CDKN1A/P21 axis maintains appropriate levels of replication factors and CDK activity during unchallenged S phase. Loss of this control mechanism deregulates origin activation and perturbs genome-wide replication fork progression. Thus, while our data support an impaired origin activation model for genetic diseases affecting CMG formation, we propose that loss of the TRP53-CDKN1A/P21 tumor suppressor axis induces inappropriate origin activation and deregulates genome-wide fork progression.

## Highlights

• Polε hypomorphic B cells show genome-wide impaired replication origin activation
• Loss of *Trp53* rescues Polε levels in *Pole4*^−/−^ cells due to increased *Pole* transcription
• TRP53-CDKN1A/P21 regulate origin activation in a CDK-inhibition-dependent manner

## Graphical abstract

**Figure:**
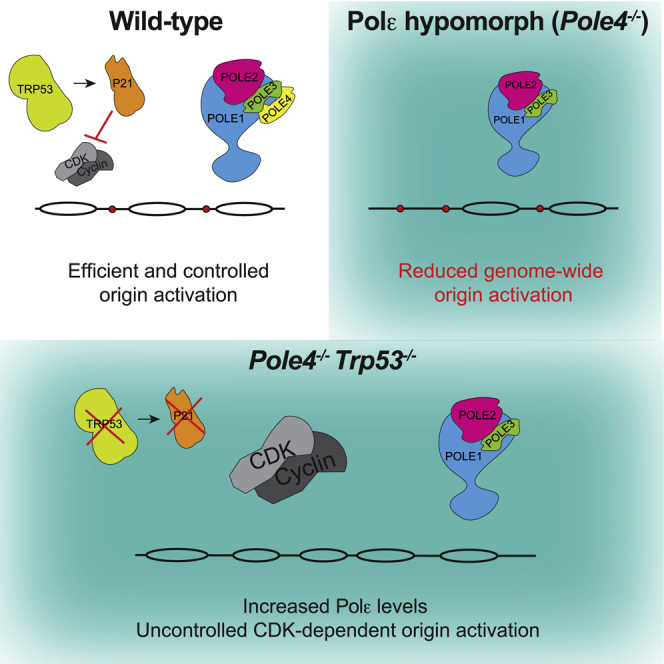

## Teaser

Borel et al. report that Polε hypomorphic cells have reduced genome-wide activation of DNA replication origins. This phenotype is rescued by the loss of TRP53 via a transcription-dependent mechanism. Furthermore, the authors found that, in primary mouse cells, the loss of TRP53/P21 induces excessive activation of DNA replication origins and replication stress.

## Introduction

DNA replication in eukaryotes is performed by a multiprotein assembly, known as the replisome, which is activated in a spatiotemporally regulated manner (Fragkos et al., 2015). At the heart of this machinery is the processive replicative helicase CMG (CDC45/MCM2–7/GINS1–4), whose establishment is regulated along the cell cycle by DDK (Dbf4-Dependent Kinase)- and CDK (Cyclin-Dependent Kinase)-dependent phosphorylation (Burgers and Kunkel, 2017). Essential components of this machinery also include the replicative polymerases Polδ and Polε, which synthesize lagging and leading strands, respectively. Importantly, Polε is also an integral component of the CMG, being required for GINS loading and formation of the pre-IC (preinitiation complex) in budding yeast (Bell and Labib, 2016).

Dysfunctional DNA replication can severely affect mammalian development and is associated with a plethora of human genetic syndromes characterized by reduced growth as well as immune and endocrine dysfunction (Bellelli and Boulton, 2021). For instance, hypomorphic mutations of the catalytic subunit of Polε, *POLE*, have been described in patients affected by FILS (facial dysmorphism, immunodeficiency, livedo, short stature) syndrome and IMAGe (intrauterine growth restriction, metaphyseal dysplasia, adrenal hypoplasia congenita, and genital anomalies in males) syndrome in association with variable degrees of immunodeficiency (Pachlopnik Schmid et al., 2012; Logan et al., 2018). Similarly, mutations of the essential non-catalytic subunit of Polε, *POLE2*, have been associated with a severe combined immunodeficiency with facial dysmorphism and impaired growth (Frugoni et al., 2016).

In addition to this, perturbed DNA replication, or replication stress, caused by oncogene activation is considered to be a major driver of genetic instability in cancer (Macheret and Halazonetis, 2015; Técher et al., 2017). In particular, dysregulated control of origin activation has been proposed to underlie oncogene-induced genetic instability in the early stages of tumorigenesis. In accordance with this, Macheret and Halazonetis recently discovered that activation of oncogenes, such as CCNE1 (Cyclin E) and MYC, induces activation of a novel set of replication origins located within highly transcribed genes, which are normally suppressed by transcription during the G1 phase of the cell cycle. Precocious G1-S transition induced by oncogene activation drives activation of these ectopic replication origins, leading to transcription-replication conflicts and genetic instability (Macheret and Halazonetis, 2018). Whether a similar mechanism is responsible for replication stress induced by loss of tumor suppressors such as P53 (TRP53 in mice) and CDKN1A/P21 remains to be established. Indeed, while E2F hyperactivation has been robustly associated with replicative stress and DNA damage (for review see Fouad et al., 2021), the role of TRP53 and CDKN1A/P21 in unchallenged DNA replication remains controversial, due to discrepancies in experimental model systems (Hampp et al., 2016; Mansilla et al., 2016; Yeo et al., 2016; Datta et al., 2017; Klusmann et al., 2016; Singh et al., 2017; Maya-Mendoza et al., 2018; Roy et al., 2018).

We previously showed that loss of the POLE4 subunit of Polε leads to a complex developmental condition in mice characterized by reduced growth, craniofacial anomalies, and lymphopenia in association with increased lymphoma predisposition (Bellelli et al., 2018a). Loss of POLE4 in mouse cells is associated with reduced levels of the POLE1 and POLE2 subunits of Polε, which led us to propose that *Pole4*^−/−^ mice might represent a Polε hypomorphic mouse model. However, more recently, we and others have also shown that POLE4 is involved in histone H3-H4 chaperoning at the replication fork (Bellelli et al., 2018b; Li et al., 2020); the consequences of the loss of this activity *in vivo* remain unclear. In addition, POLE4, in concert with POLE3, is also a component of the acetyltransferase complex ATAC (Wang et al., 2008), and its deficiency has been recently shown to promote sensitivity to both ATR and PARP inhibitors (Hustedt et al., 2019; Su et al., 2020). While the mechanism behind this phenomenon remains unknown, insights into this process may lead to the identification of new markers of sensitivity to these compounds and novel vulnerabilities of cancer cells.

Here we show that Polε instability, caused by the loss of POLE4, drives reduced replication origin activation in primary B cells, independent of their genomic location. Surprisingly, the phenotypic consequences of Polε instability are rescued by TRP53 depletion in mice and cells. While the lack of POLE4 drives proteasomal-dependent degradation of Polε, loss of TRP53 in Polε hypomorphic cells restores “close to wild-type” levels of Polε, due to increased transcription of Polε subunits. Through the analysis of the transcriptome and replication dynamics of *Trp53* knockout cells, we then discovered that genetic deletion of *Trp53* leads to suppression of *Cdkn1a/p21* in primary mouse cells and a concomitant increase in E2F activity and replication origin activation, in association with an increased level of replication initiation factors. Hyperactivation of DNA replication origins upon dysregulation of the TRP53-CDKN1A/P21 axis depends on the CDK inhibitory domain of CDKN1A/P21 and leads to genome-wide perturbed replication fork progression. This mechanism has broad consequences for genetic instability caused by loss of the TRP53 and CDKN1A/P21 tumor suppressors and therapeutic targeting of cancer cells.

## Results

### Loss of *Pole4* leads to genome-wide reduced initiation of DNA replication in primary B cells

Genetic ablation of *Pole4* in mice leads to a multifaceted disorder characterized by reduced growth, developmental abnormalities, lymphopenia, and increased lymphomagenesis, which resembles IMAGe syndrome in patients affected by hypomorphic mutations of *POLE* (Logan et al., 2018; Bellelli et al., 2018a). Interestingly, both *Pole4*^−/−^ MEFs (mouse embryo fibroblasts) and *POLE* mutant patient-derived cells exhibit increased interorigin distance, suggestive of disrupted replication origin control. How replication origins are activated genome-wide in these pathological conditions had not been explored, but might reveal the mechanistic basis of diseases caused by mutation of CMG components (Bellelli and Boulton, 2021).

**Figure 1:**
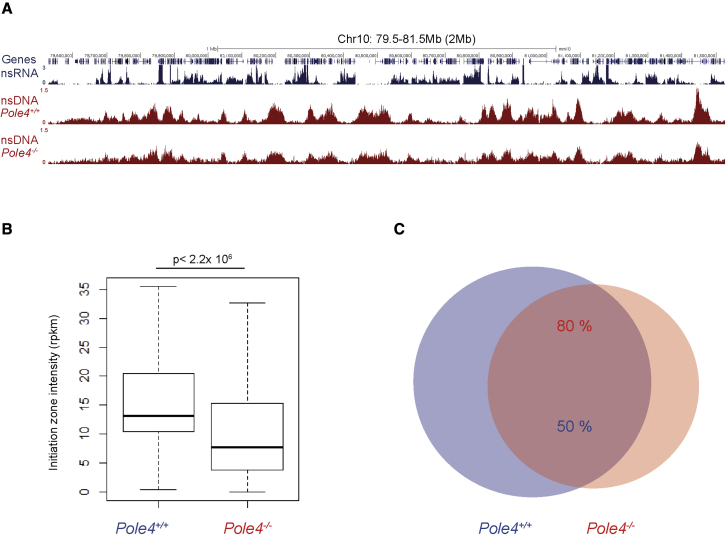
*Pole4*^−/−^ primary B cells show reduced genome-wide replication origin activation (A) Genome browser screenshot displaying nascent RNA-seq (blue) and HU-EdU-seq (red) profiles shown as normalized read density (reads per million, RPM) for *Pole4*^+/+^ and *Pole4*^−/−^ primary B cells upon 28 h stimulation by LPS/IL-4. (B) Quantification of HU-EdU-seq signal (reads per kilobase per million, RPKM) at replication initiation zones in *Pole4*^+/+^ (n = 10,299) and *Pole4*^−/−^ (n = 6,432) primary B cells. (C) Venn diagram showing overlap between initiation zones in *Pole4*^+/+^ (n = 10,299) and *Pole4*^−/−^ (n = 6,432) primary B cells.

To map sites of replication initiation in *Pole4*^−/−^ mice, we conducted HU-EdU-seq (hydroxyurea-EdU sequencing) at high resolution in primary mouse cells (Tubbs et al., 2018). To this end, we isolated primary B cells from *Pole4*^+/+^ and *Pole4*^−/−^ mouse spleens and activated them with LPS/IL-4 (lipopolysaccharide/interleukin-4) to ensure synchronous cell-cycle entry. More specifically, cells were labeled with EdU (20 μM) in the presence of 10 mM HU, and nsDNA (nascent-strand DNA) was isolated by Click chemistry and subjected to high-throughput sequencing as previously described (Tubbs et al., 2018). In accordance with our previous findings, initiation zones were strongly enriched between transcribed genes in early replicating regions in both *Pole4*-deficient and proficient cells (Figure 1A and Tubbs et al., 2018). Strikingly, this analysis also revealed that replication initiation events were strongly reduced in *Pole4*^−/−^ cells compared with wild type (p < 2.2 × 10^−16^) (Figures 1A and 1B). Importantly, while the overall numbers of replication initiation events were strongly reduced in *Pole4*-deficient cells, the sites of initiation greatly overlapped between WT (wild-type) and KO (knockout) cells (Figure 1C), thus suggesting an overall reduced efficiency of initiation, independent of replication origin location in the genome.

### Genetic deletion of *Trp53* increases Polε subunit levels in *Pole4*^−/−^ mouse cells

**Figure 2:**
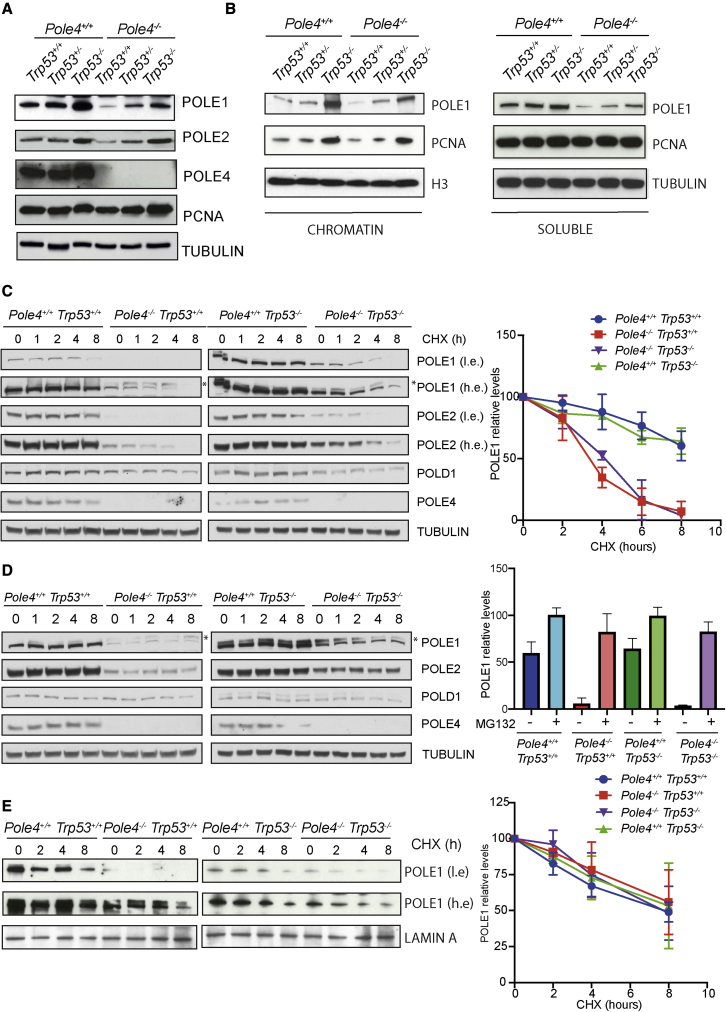
Genetic deletion of *Trp53* rescues Polε subunit levels in *Pole4*^−/−^ cells in a proteasome-independent manner (A) Western blot analysis of Polε subunits and PCNA from total extracts of *Pole4*^+/+^ and *Pole4*^−/−^ MEFs in a *Trp53* WT, HET, or KO background. Tubulin was used for normalization. (B) Western blot analysis of POLE1 and PCNA in the soluble and chromatin fractions of the described MEF genotypes. Tubulin and histone H3 were used as loading controls. (C) Left: western blot analysis of Polε subunits and POLD1 from total extracts of *Pole4*^+/+^ and *Pole4*^−/−^ MEFs in a *Trp53* WT (left blot) or KO (right blot) background, treated with CHX (cycloheximide) for the indicated time points (h). Tubulin was used for normalization. ^∗^Non-specific band in the POLE1 western blot (see also Figure S3C). Right: half-life curve of POLE1 protein levels in MEFs of the described genotypes, incubated with CHX for the indicated time points. Results are reported as the mean ± SD of triplicate experiments. The lower and specific band in the POLE1 western blot was used for quantification. (D) Left: western blot analysis of Polε subunits and POLD1 from total extracts of *Pole4*^+/+^ and *Pole4*^−/−^ MEFs in a *Trp53* WT (left blot) or KO (right blot) background, treated with CHX and MG132 for the indicated time points. Tubulin was used for normalization. ^∗^Non-specific band in the POLE1 western blot (see also Figure S3C). Right: bar graph showing POLE1 relative levels in MEFs of the described genotype incubated for 8 h with CHX, in the presence or not of MG132. POLE1 levels were normalized to those of untreated cells and results are reported as the mean ± SD of triplicate experiments. The lower and specific band in the POLE1 western blot was used for quantification. (E) Left: western blot analysis of POLE1 levels in the chromatin fraction of *Pole4*^+/+^ and *Pole4*^−/−^ MEFs in a *Trp53* WT (left blot) or KO (right blot) background, treated with CHX for the indicated time points (h). Lamin A was used for normalization. Right: half-life curve of POLE1 chromatin levels in MEFs of the described genotypes, incubated with CHX for the indicated time points. Results are reported as the mean ± SD of triplicate experiments.

The loss of both copies of the tumor suppressor *Trp53* rescued embryonal lethality and phenotypical anomalies observed in *Pole4*^−/−^ mice in the C57BL/6 genetic background (Bellelli et al., 2018a). Interestingly, C57BL/6 *Pole4*^−/−^ *Trp53*^+/−^ mice present with an intermediate phenotype with congenital anomalies similar to those observed in *Pole4*^−/−^ animals in a mixed genetic background. These observations prompted us to evaluate the interplay between POLE4 and TRP53 in the control of DNA replication and genome stability. To this end, we initially analyzed MEFs from *Pole4*^+/+^ and *Pole4*^−/−^ embryos in a *Trp53* WT or KO genetic background. As previously shown, *Pole4*^−/−^ primary mouse cells showed a strong reduction in the levels of the POLE1 and, to a lesser extent, POLE2 subunits of Polε, compared with WT cells (Figures S1A and S2A). However, to our surprise, we discovered that *Pole4*^−/−^ *Trp53*^−/−^ primary cells showed expression levels of Polε subunits close to those of WT, suggesting a rescue of Polε stability (Figures S1A and S2A). The presence of an intermediate phenotype in *Pole4*^−/−^ *Trp53*^+/−^ mice remained to be explained. Thus, we established MEFs from *Pole4* WT and KO animals in *Trp53*^+/+^, *Trp53*^+/−^, and *Trp53*^−/−^ backgrounds and observed that *Pole4*^+/+^ *Trp53*^+/−^ MEFs showed intermediate levels of POLE1 and POLE2 compared with WT and double-null cells, pointing to a dose-dependent control by TRP53 of Polε stability in *Pole4*-deficient cells (Figure 2A). A similar increase in Polε subunits levels was observed in large-T-immortalized MEFs from *Pole4*^−/−^ embryos, suggesting a TRP53-specific function in controlling Polε complex levels (Figure S1B). Intriguingly, increased overall expression levels of Polε subunits also led to increased chromatin levels of POLE1 and restored levels of PCNA on chromatin, suggesting a rescue of DNA replication origin activation (Figures 2B and S1C). Importantly, CDC7 kinase inhibition abolished chromatin binding of Polε and PCNA, pointing to a replication-initiation-dependent mechanism (Figure S1D). In addition to this, cell-cycle flow cytometry showed that the increased POLE1 signal in the absence of TRP53 was not a consequence of an increased percentage of S-phase cells (Figures S2A and S2B).

### Loss of POLE4 leads to proteasome-dependent Polε degradation, which is not affected by depletion of TRP53

To investigate the mechanism responsible for reduced levels of Polε complex subunits upon loss of *Pole4* and its rescue upon ablation of *Trp53*, we incubated *Pole4*^+/+^ and *Pole4*^−/−^ MEFs with cycloheximide, an inhibitor of protein synthesis, and assessed the levels of POLE1 and POLE2 by western blotting. As shown in Figure 2C, in WT mouse cells, the half-life of POLE1 and POLE2 was 8 h or longer (Figure 2C, left and half-life curve). In contrast, in *Pole4*^−/−^ cells, the half-life of Polε complex subunits was reduced to less than 4 h, suggesting that interaction with POLE4 is required in mouse cells to maintain the stability of the whole Polε complex. The half-life of other DNA polymerase subunits, such as POLD1, the major and catalytic subunit of Polδ, was not affected, excluding a non-specific effect (Figure 2C, left). To investigate a possible role for TRP53 in regulating Polε complex stability, we then performed cycloheximide pulse labeling in *Pole4*^+/+^ *Trp53*^−/−^ and *Pole4*^−/−^ *Trp53*^−/−^ MEFs and analyzed the levels of POLE1 and POLE2 by western blotting. Despite the fact that *Trp53*^−/−^ cells showed increased levels of Polε complex subunits, a lack of *Trp53* did not affect the half-life of POLE1 and POLE2 in a *Pole4*^−/−^ background (Figure 2C, right and half-life curve). Importantly, degradation of POLE1 and POLE2 was inhibited upon treatment with the proteasome inhibitor MG132, which suggests that the POLE1-POLE2 subcomplex is targeted for proteasome-dependent degradation in the absence of POLE4, in a TRP53-independent manner (Figure 2D).

We considered the possibility that Polε degradation might occur before or after its engagement in DNA replication. Indeed, POLE3-POLE4, and their yeast ancestors, bind dsDNA *in vitro*, which suggests they might be required for efficient engagement of Polε with DNA at the replication fork (Bellelli et al., 2018b; Tsubota et al., 2006). To evaluate the stability of Polε in the chromatin compartment, we pulse labeled *Pole4*^+/+^ and *Pole4*^−/−^ MEFs, in a *Trp53* WT or KO background, with cycloheximide and performed western blotting analysis of POLE1 levels in the soluble and chromatin fractions. Strikingly, the half-lives of POLE1 on chromatin were similar between *Pole4*^+/+^ and *Pole4*^−/−^ MEFs, in the presence or absence of both *Trp53* copies (Figure 2E). In contrast, the levels of the catalytic subunit of Polε were significantly reduced in a *Pole4*-null background in the soluble fraction, which likely represents newly synthesized POLE1, not yet engaged in DNA replication (Figure S2C). Thus, once engaged with the CMG in chromatin DNA replication, Polε is sufficiently stable in the presence or absence of POLE4.

### Loss of TRP53 leads to increased expression levels of DNA replication genes

**Figure 3:**
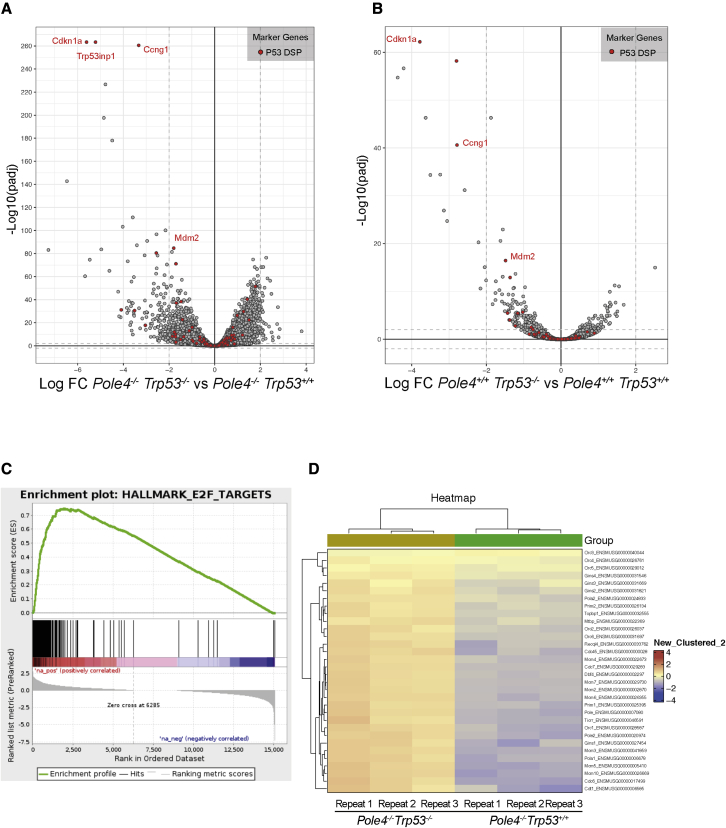
Transcriptomic analysis of *Pole4*^−/−^ cells in a *Trp53* WT and KO genetic background (A) Volcano plot of RNA-seq analysis displaying gene expression values for *Pole4*^−/−^*Trp53*^−/−^ relative to *Pole4*^−/−^*Trp53*^+/+^ MEFs. The x axis represents the log_2_ fold change, while the y axis represents the negative decade logarithm of the significance value change. Red dots indicate annotated *Trp53* downstream targets; *Cdkn1a/p21*, *Trp53inp1*, *Ccng1*, and *Mdm2* are indicated among those. (B) Volcano plot of RNA-seq analysis displaying gene expression values for *Pole4*^+/+^*Trp53*^−/−^ relative to *Pole4*^+/+^*Trp53*^+/+^ MEFs. The x axis represents the log_2_ fold change, while the y axis represents the negative decade logarithm of the significance value change. Red dots indicate annotated *Trp53* downstream targets. (C) GSEA (gene set enrichment analysis) plot of genes enriched in *Pole4*^−/−^*Trp53*^−/−^ versus *Pole4*^−/−^*Trp53*^+/+^. The green line indicates the enrichment score along the ranked gene set, while the dark lines indicate the positions of genes in the ranked gene list. (D) Heatmap of genes required for the initiation of DNA replication from triplicate RNA-seq of the indicated MEF genotypes.

Having established that loss of TRP53 does not rescue Polε levels by promoting its stability, we next tested the effect of TRP53 loss on the transcriptional program of *Pole4*^+/+^ and *Pole4*^−/−^ cells. To evaluate the transcriptome of *Pole4*-proficient and -deficient cells, in the presence or absence of one or two copies of *Trp53*, we performed RNA-seq (RNA-sequencing) experiments from *Pole4*^+/+^ and *Pole4*^−/−^ MEFs that were *Trp53*^+/+^, *Trp53*^+/−^, or *Trp53*^−/−^*.* We then generated a volcano plot of fold expression change comparing *Pole4*^−/−^ *Trp53*^+/+^ versus *Pole4*^−/−^ *Trp53*^−/−^ MEFs. Interestingly, among the well-characterized TRP53 targets (highlighted in red in Figure 3A), only a few were significantly affected in primary mouse cells (Pfister and Prives, 2017; Fischer, 2017). The most downregulated gene in *Pole4*^−/−^ *Trp53*^−/−^ cells was *Cdkn1a*/p21, a well-known TRP53 target and an inhibitor of G1-S and S-phase cyclin-CDK complexes (Abbas and Dutta, 2009). Other previously identified TRP53 targets such as *Trp53inp1* and *Cyclin G1* were also strongly downregulated (Fischer, 2017, and Figure 3A). Similar results were obtained by comparing expression changes of *Pole4*^+/+^ *Trp53*^+/+^ versus *Pole4*^+/+^ *Trp53*^−/−^ cells, with *Cdkn1a*/p21 again being the most affected gene (Figure 3B). Hence, the loss of TRP53 affects the expression levels of a core set of genes in primary MEFs in the presence or absence of *Pole4*.

Importantly, our RNA-seq experiments were performed in passage 1 primary MEFs grown in low oxygen concentrations, suggesting that low levels of TRP53 normally sustain regulated gene expression along an unperturbed cell cycle. Our analysis also suggested that CDKN1A/P21 might represent the main factor required for this control. Indeed, by controlling Cyclin-CDK activity at the G1-S transition and Cyclin A-CDK2 during S phase, CDKN1A/P21 might affect the E2F transcriptional program and DNA replication (Kent and Leone, 2019). Accordingly, most upregulated genes in *Pole4*^−/−^ *Trp53*^−/−^ as well as in *Pole4*^+/+^ *Trp53*^−/−^ MEFs are well-known E2F target genes, as visualized in the volcano plot and in a GSEA (gene set enrichment analysis) of E2F targets (Figures 3C, S3A, and S3B). Importantly most of the genes upregulated in *Trp53*^−/−^ cells (previously annotated or not in the E2F signature) are involved in the control of DNA replication initiation (Figure 3D). Accordingly, both POLE1 and POLE3 subunits of Polε have been previously reported to be regulated by E2F-dependent promoters (Huang et al., 1999; Bolognese et al., 2006). Thus, increased expression levels of *Pole* are not a specific feature of *Trp53*-null cells and likely depend upon a common E2F signature downstream of deregulated *Cdkn1a/p21* levels. Accordingly, transient siRNA (small interfering RNA)-mediated knockdown of *Cdkn1a/p21* strongly increased POLE1 and POLE2 levels in both *Pole4*^+/+^ and *Pole4*^−/−^ cells (Figure S3C). Furthermore, a significant increase in POLE1 levels was also observed upon transient retroviral-mediated expression of E2F1 (Figure S3D).

All together, our data suggest that the absence of TRP53 leads to reduced basal expression levels of CDKN1A/P21 during an unperturbed S phase, which drives increased Cyclin-CDK activity and E2F-dependent expression of genes required for initiation of DNA replication.

### Loss of TRP53 leads to increased cell growth and reduced DNA damage in *Pole4*^−/−^ cells

**Figure 4:**
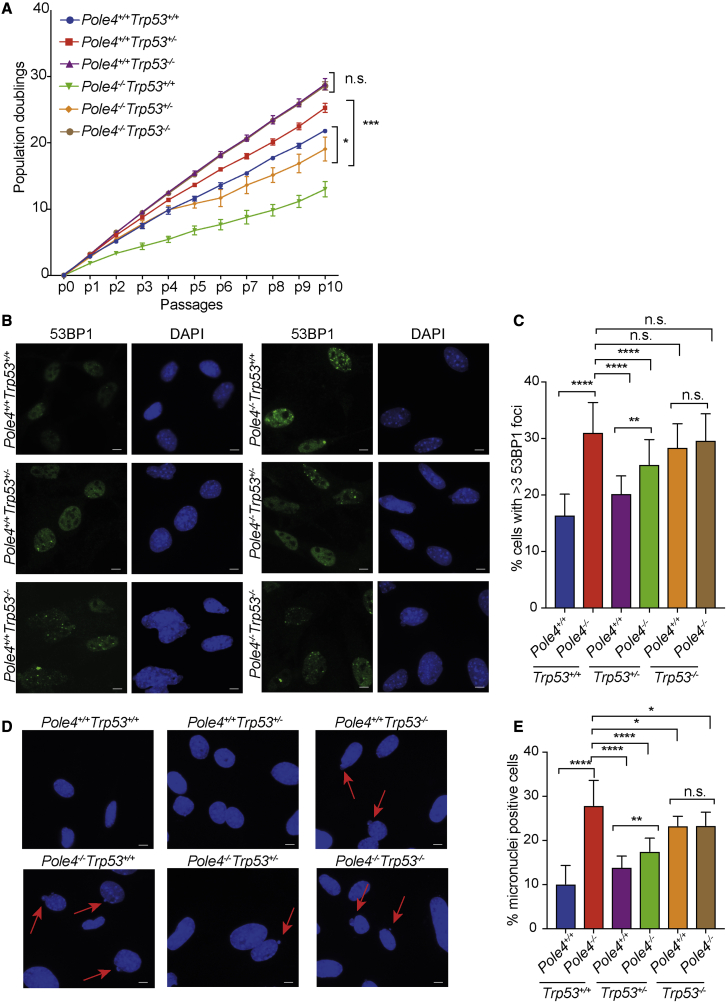
Loss of *Trp53* rescues growth rate and DNA damage accumulation in *Pole4*^−/−^ cells (A) Population doubling accumulation in primary MEFs from the indicated genotypes. Cells were cultured according to a standard 3T3 protocol; unpaired t test analysis: ^∗^p < 0.05; ^∗∗∗^p < 0.001; n.s., not significant. Results are reported as the mean ± SD of triplicate experiments. (B) Representative pictures from immunofluorescence staining for 53BP1 in primary MEFs of the indicated genotypes. (C) Bar graph showing the percentage of cells, in the indicated genotypes, with more than three 53BP1 foci; unpaired t test analysis: ^∗∗^p < 0.01, ^∗∗∗∗^p < 0.0001. Results are reported as the mean ± SD of six different experiments (two different slides from three different biological replicates). (D) Representative pictures of micronuclei from cells of the indicated genotypes. (E) Bar graph showing the percentage of micronuclei-positive cells in the indicated genotypes; unpaired t test analysis: ^∗^p < 0.05, ^∗∗^p < 0.01, ^∗∗∗∗^p < 0.0001. Results are reported as the mean ± SD of six different experiments (two different slides from three different biological replicates). All scale bars represent 10 μm.

To further understand the contribution of a loss of TRP53 and Polε instability to the phenotypes we observed in *Pole4*-null cells and mice, we proceeded to analyze growth rate and markers of DNA damage in *Pole4*^+/+^ and *Pole4*^−/−^ MEFs in *Trp53*^+/+^, *Trp53*^+/−^, and *Trp53*^−/−^ backgrounds. *Pole4*^−/−^ primary cells grown in low oxygen concentrations showed reduced proliferation potential as shown by cumulative population-doubling analysis. This was particularly evident in the C57BL/6 background, in which the *Pole4*-null allele results in late embryonic lethality (Figure 4A and Bellelli et al., 2018a). Interestingly, in a heterozygous *Trp53* background, *Pole4*^−/−^ cells showed an increased replication potential compared with *Trp53* WT cells; however, the overall number of population doublings was still reduced compared with *Pole4*^+/+^ *Trp53*^+/−^ cells, which is suggestive of a partial rescue of proliferation rates (Figure 4A). These data are in accordance with the fact that *Pole4*^−/−^ *Trp53*^+/−^ mice show an intermediate phenotype in the C57BL/6 background (Bellelli et al., 2018a). In contrast, *Pole4*^−/−^ *Trp53*^−/−^ cells exhibited a strong proliferative growth and were indistinguishable from *Pole4*^+/+^ *Trp53*^−/−^ cells, which suggests a complete restoration of proliferation upon loss of both copies of *Trp53* (Figure 4A). In agreement with a CDKN1A/P21-dependent mechanism, transient siRNA-mediated knockdown of CDKN1A/P21 restored the proliferation rates of *Pole4*^−/−^ cells, as shown by population doubling accumulation (Figure S4A).

We initially hypothesized that a loss of TRP53 might rescue the phenotypical abnormalities of *Pole4*-null mice by allowing proliferation of genetically unstable cells (Bellelli et al., 2018a). However, the puzzling absence of increased tumorigenesis in *Pole4*^−/−^ *Trp53*^−/−^ mice remained unexplained. With this in mind, we stained primary MEFs in the different genetic backgrounds for markers of DNA damage, such as 53BP1, and analyzed the numbers of micronuclei per cell. In accordance with reduced growth rates being dependent on DNA damage accumulation, *Pole4*^−/−^ cells showed a strong increase in the percentage of 53BP1 and micronuclei-positive cells (Figures 4B, 4C, 4D, and 4E). Markers of DNA damage were reduced in a *Pole4*^−/−^ *Trp53*^+/−^ cells compared with *Pole4*^−/−^ *Trp53*^+/+^, but significantly increased compared with *Pole4*^+/+^ *Trp53*^+/−^ cells, again suggestive of a partial suppression of DNA damage and genome instability. In addition to this, while *Pole4*^+/+^ *Trp53*^−/−^ showed an increased accumulation of DNA damage compared with WT cells, as seen by both 53BP1 foci and micronuclei accumulation, this was not statistically different from *Pole4*^−/−^ *Trp53*^−/−^ (Figures 4B, 4C, 4D, and 4E).

Finally, to confirm that the phenotypic rescue observed in *Pole4*^−/−^ cells upon deletion of *Trp53* is caused by increased Polε levels, we infected primary MEFs with retroviral vectors expressing the catalytic subunit of Polε, POLE1 (Figure S4B). In accordance with this hypothesis, expression of POLE1 alone rescued the cellular proliferation of *Pole4*^−/−^ MEFs, as shown by population doubling accumulation (Figure S4C). Taken together, these data suggest that the loss of both copies of *Trp53* suppresses the accumulation of DNA damage caused by the absence of POLE4, thus rescuing the cellular proliferation of *Pole4*^−/−^ cells, in a Polε-level-dependent manner.

### DNA replication dynamics upon loss of POLE4 and TRP53

**Figure 5:**
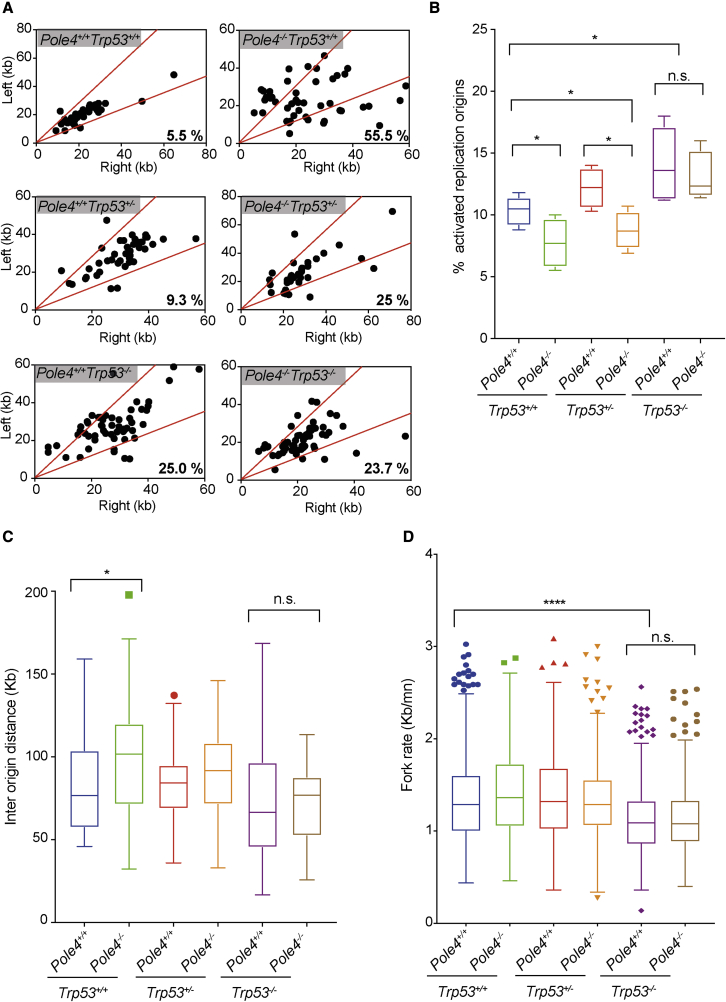
Analysis of replication fork dynamics in *Pole4*^+/+^ or ^−/−^ cells in a Trp53 WT, HET, or KO genetic background (A) Analysis of replication fork symmetry in *Pole4*^+/+^ and *Pole4*^−/−^ MEFs in a *Trp53* WT, HET, or KO background, reported as left/right moving forks ratio. (B) Graph showing the percentage of newly activated replication forks in *Pole4*^+/+^ and *Pole4*^−/−^ MEFs in a *Trp53* WT, HET, or KO genetic background; unpaired t test analysis: ^∗^p < 0.05; n.s., not significant. (C) Graph showing the mean interorigin distance in the described genetic backgrounds; unpaired t test analysis: ^∗^p < 0.05; n.s., not significant. (D) Graph showing replication fork elongation rate in *Pole4*^+/+^ and *Pole4*^−/−^ MEFs in a *Trp53* WT, HET, or KO genetic background; unpaired t test analysis: ^∗∗∗∗^p < 0.0001; n.s., not significant. Fiber experiments were performed four times and results are reported as box-and-whiskers plots using the Tukey method.

To understand the contribution of Polε instability and TRP53 to the control of origin activation and replication fork progression, we went on to investigate the dynamics of DNA replication in *Pole4*-proficient and -deficient cells, in the presence or absence of one or two copies of *Trp53*. We first analyzed the presence of asymmetric replication forks, as a marker of fork stalling events and replication stress (Técher et al., 2017). *Pole4*^−/−^ *Trp53*^+/+^ cells showed a remarkably high percentage of fork asymmetry (Figure 5A). Interestingly, *Pole4*^+/+^ *Trp53*^−/−^ cells also presented with increased fork asymmetry compared with WT cells, albeit to a lower extent than that observed in *Pole4*-deficient cells (Figure 5A). Strikingly, *Pole4*^−/−^ *Trp53*^−/−^ MEFs showed levels of fork asymmetry similar to those of *Pole4*^+/+^ *Trp53*^−/−^ cells, suggesting that the loss of TRP53 in a *Pole4*-null background is associated with a “relative” rescue of replication stress. We then analyzed the overall number of newly active replication origins. While *Pole4*-deficient cells showed a reduction in the number of replication initiation events, the percentage of newly activated replication forks was increased in both *Pole4*^+/+^ *Trp53*^−/−^ and *Pole4*^−/−^ *Trp53*^−/−^ cells compared with *Pole4*-proficient and -deficient cells in a *Trp53* WT background (Figures 5B and S5A). While pointing to increased origin activation in a *Trp53-null* genetic background, these data are consistent with a rescue of replication origin activation in *Pole4*^−/−^ cells upon loss of TRP53. Accordingly, *Pole4*^−/−^ *Trp53*^−/−^ and, to a larger extent, *Pole4*^+/+^ *Trp53*^−/−^ cells presented with reduced interorigin distances (Figure 5C). Moreover, and consistent with increased replication origin activation and replication factor consumption, both *Pole4*^+/+^ *Trp53*^−/−^ and *Pole4*^−/−^ *Trp53*^−/−^ MEFs showed reduced fork speed (Figure 5D). Finally, in agreement with a limiting role for POLE1 in origin activation, in *Pole4*^−/−^ cells, overexpression of POLE1 not only rescued cellular proliferation, but also rescued origin activation, as shown by the “normalization” of interorigin distance values (Figure S5B). Collectively these data suggest that loss of TRP53 increases origin activation and promotes a relative rescue of replication dynamics in *Pole4*^−/−^ cells.

### CDC7 inhibition rescues reduced fork speed in *Trp53*^−/−^ cells

**Figure 6:**
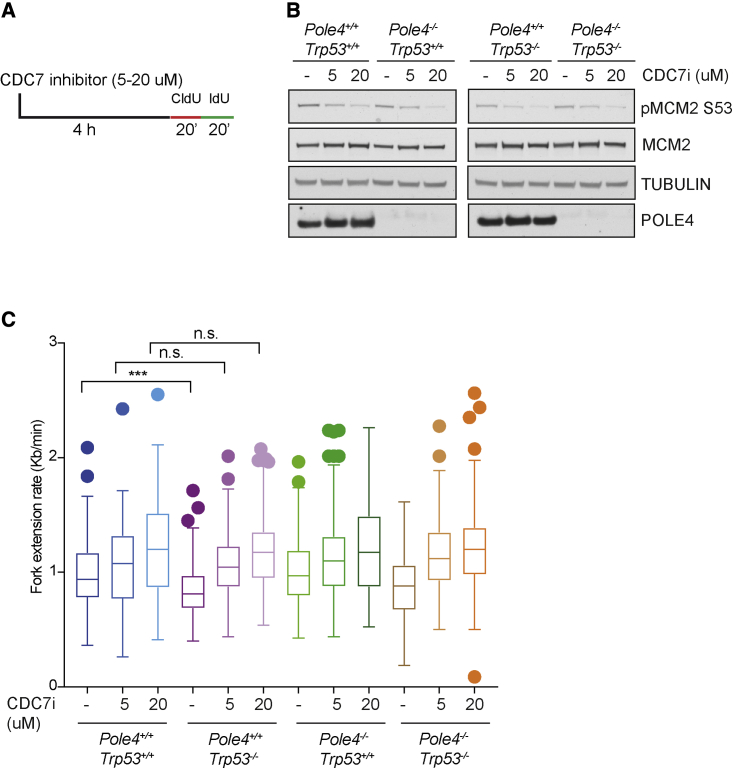
Analysis of replication fork dynamics upon CDC7 pharmacological inhibition (A) Scheme of the CDC7 inhibition and CldU/IdU labeling used for experiments shown in (B) and (C). (B) Western blot analysis of MCM2 phosphorylation levels on Ser53 in *Pole4*^+/+^ and MEFs, in a *Trp53* WT or KO background, treated with the indicated doses of the CDC7 inhibitor PHA-767491. (C) Graph showing replication fork elongation rates in *Pole4*^+/+^ and *Pole4*^−/−^ MEFs in a *Trp53* WT or KO genetic background upon treatment with the CDC7 inhibitor PHA-767491 at 5 or 20 μM; unpaired t test analysis: ^∗∗∗^p < 0.001; n.s., not significant. Fiber experiments were performed four times and results are reported as box-and-whisker plots using the Tukey method.

We considered that the decreased fork speed and interorigin distance observed upon genetic ablation of *Trp53* might depend on a primary defect in replication fork progression or may represent a compensatory effect induced by excessive origin activation, similar to that observed upon oncogene activation *in vitro* (Técher et al., 2017). Previous work has shown that oncogene activation induces inappropriate origin activation, nucleotide depletion, and a compensatory decrease in fork speed (Bester et al., 2011). To distinguish between these possibilities, we transiently inhibited origin activation with a specific CDC7 inhibitor, PHA-767491, and analyzed fork speed under the previously described genetic conditions (Montagnoli et al., 2008). To this end, we treated *Pole4*^+/+^ and *Pole4*^−/−^ cells in a *Trp53*-proficient and -deficient background with 5 and 20 μM PHA-767491 for 4 h, and then labeled them with two consecutive pulses of CldU and IdU in the presence of the same drug concentration (Figure 6A). In accordance with previous data and PHA-767491 activity in our cells, CDC7 inhibition reduced phosphorylation of MCM2 on Ser53 in a *Trp53-* and *Pole4*-independent manner (Montagnoli et al., 2008; Figure 6B). We then analyzed fork speed under all the described genetic conditions and drug concentrations. As shown in Figure 6C, inhibition of CDC7 with 5 μM and, to a higher extent, 20 μM PHA-767491 caused a significative increase in fork speed in both *Trp53*-proficient and -deficient cells; thus, replication forks are capable of traveling fast in the absence of TRP53, consistent with origin activation being the principal step affected by TRP53 loss in the presence and absence of POLE4.

### Transient or genetic knockdown of *Cdkn1a/p21* reproduces replication dynamics of *Trp53*^−/−^ primary mouse cells

DNA fiber analysis in primary MEFs suggests that a loss of TRP53 might alter origin activation, secondarily causing dysfunctional genome-wide fork progression and genome instability. Our transcriptome analysis in *Trp53*^−/−^ cells points to the loss of *Cdkn1a/p21* expression as the strongest candidate to explain this phenomenon. Despite this, the loss of *Trp53* also causes a reduction in the levels of its negative regulator *Mdm2*, which has been identified as a potential modulator of DNA replication (Klusmann et al., 2016).

To test the involvement of CDKN1A/P21 and MDM2 in this process, we initially transfected WT primary MEFs with siRNAs targeting *Cdkn1a/p21, Mdm2*, or a control sequence and monitored replication fork speed as a measure of replicative stress. Preliminarily, we were able to detect CDKN1A/P21 and MDM2 expression and their downregulation upon siRNA transfection in early passage (passage 1) primary MEFs, grown under low oxygen conditions (Figure S6A).

**Figure 7:**
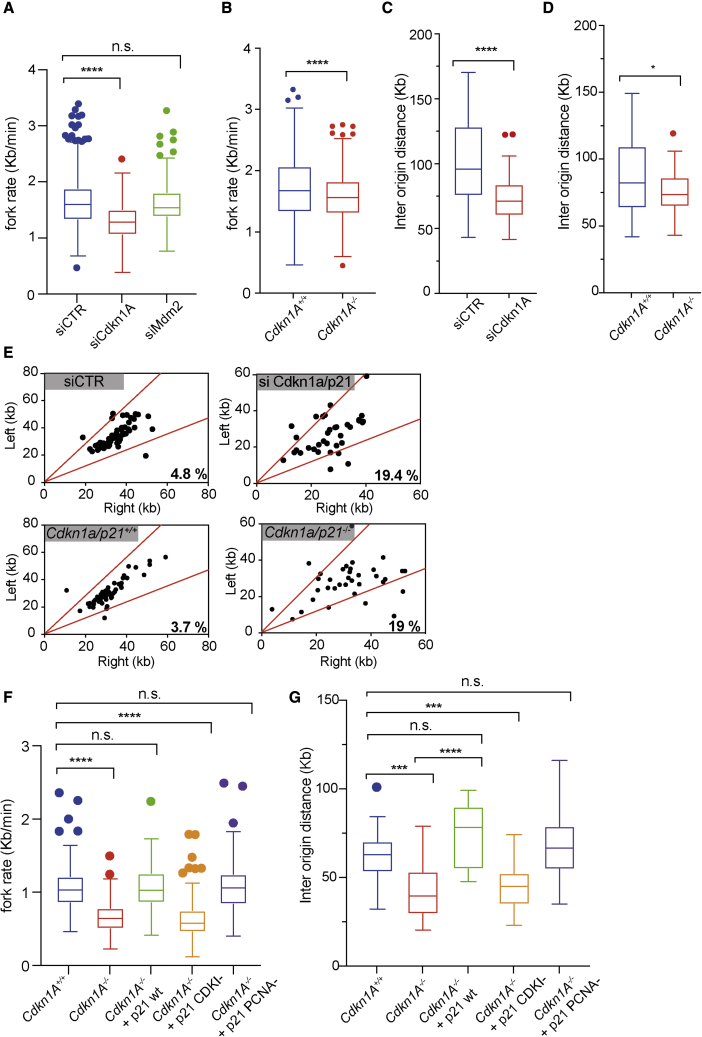
Analysis of replication fork dynamics upon loss of CDKN1A/P21 and its CDK inhibitory and PCNA binding domains (A) Graph showing replication fork elongation rates in primary MEFs transfected with siRNAs against *Cdkn1a/p21*, *Mdm2*, or control siRNA. (B) Graph showing replication fork elongation rates of primary MEFs from *Cdkn1a/p21*^+/+^ and *Cdkn1a/p21*^−/−^ mice. (C) Graph showing IOD (interorigin distance) values in primary MEFs transfected with siRNAs against *Cdkn1a/p21* or control siRNAs. (D) Graph showing IOD values in primary MEFs from *Cdkn1a/p21*^+/+^ or *Cdkn1a/p21*^−/−^ mice. (E) Analysis of replication fork symmetry in primary *Cdkn1a/p21*^+/+^ or *Cdkn1a/p21*^−/−^ MEFs or cells transfected with siRNAs against *Cdkn1a/p21* or control siRNA. Data are reported as left/right moving forks ratio. (F) Graphs showing replication fork elongation rates of primary *Cdkn1a/p21*^−/−^ MEFs infected with retroviruses expressing CDKN1A/P21 WT or mutants unable to inhibit CDKs (CDKI^−^) or bind PCNA (PCNA^−^). (G) Graph showing IOD values of primary MEFs infected with retroviruses expressing CDKN1A/P21 WT or mutants unable to inhibit CDKs (CDKI^−^) or bind PCNA (PCNA^−^); unpaired t test analysis: ^∗^p < 0.05; ^∗∗∗^p < 0.001; ^∗∗∗∗^p < 0.0001; n.s., not significant. Fiber experiments were performed four times and results are reported as box-and-whiskers plots using the Tukey method.

Strikingly, transient knockdown of *Cdkn1a/p21*, but not *Mdm2*, led to a strong decrease in fork speed, mimicking the phenotype observed in *Trp53*^−/−^ primary cells (Figure 7A). Similar results were obtained in primary MEFs harboring a genetic KO of *Cdkn1a/p21* (Figure 7B). Importantly, reduced fork speed was associated with increased fork asymmetry and shorter interorigin distances, suggestive of increased origin activation, upon both transient and stable genetic KO of *Cdkn1A/p21* (Figures 7C, 7D, and 7E).

CDKN1A/P21 was initially discovered as a universal CDK inhibitor (Harper et al., 1993; Xiong et al., 1993). Subsequent studies identified a C-terminal PCNA-interacting protein domain (or PIP box) involved in DNA synthesis and repair transactions (Waga et al., 1994; Chen et al., 1995; for review see Cazzalini et al., 2010). To dissect the contributions of these separate activities, we infected primary *Cdkn1A/p21* KO MEFs with retroviral vectors expressing WT CDKN1A/P21 or mutants unable to bind or inhibit CDKs (CDKI^−^) or PCNA (PCNA^−^) (Figure S7B) (Cayrol et al., 1998; Abbas et al., 2008). Strikingly, expression of close to endogenous levels of CDKN1A/P21 WT, but not its CDKI^−^ mutant, rescued both fork speed and interorigin distance levels (Figures 7F and 7G). Importantly, similar to CDKN1A/P21 WT, complementation of *Cdkn1A/p21*-deficient cells with the PCNA binding mutant rescued both reduced fork speed and interorigin distance values (Figures 7F and 7G). Thus, loss of control of CDK activity during S phase is the primary mechanism that perturbs replication fork dynamics in primary mouse cells upon loss of CDKN1A/P21.

All together, our data strongly suggest that loss of the TRP53-CDKN1A/P21 axis disrupts the CDK-dependent control of origin activation, driving altered genome-wide fork progression in primary mammalian cells.

## Discussion

Here we show that Polε and TRP53 levels are crucial for the control of DNA replication origin activation and the maintenance of genome stability in mammals. Analysis of primary B cells revealed that lack of the POLE4 subunit of Polε impairs genome-wide activation of DNA replication origins due to destabilization and proteasome-dependent degradation of Polε, pointing to a hypomorphic mechanism. Unexpectedly, we found that loss of TRP53 in *Pole4*-deficient cells rescued replication origin activation and DNA damage accumulation by restoring Polε protein levels. Our data suggest that the TRP53-CDKN1A/P21 axis finely tunes replication factor levels and origin activation to ensure accurate and efficient genome duplication during the S phase of the cell cycle.

Disruption of *Pole4* in mice confers a Polε hypomorphic phenotype, which manifests as impaired replication origin activation *in vivo*. In budding yeast, Polε is required for GINS loading at replication origins and establishment of the CMG helicase; this is followed by recruitment of additional replication factors and origin firing (Bell and Labib, 2016). Our HU-EdU-seq, chromatin purification, and DNA fiber experiments clearly showed a reduction in origin activation in Polε hypomorphic cells. While we speculate that reduced levels of Polε might compromise formation of the CMG complex, it is possible that origin activation is affected at a later stage, such as activation of the helicase activity of the CMG. Consistent with a Polε hypomorphic mechanism, overexpression of POLE1, the catalytic subunit of Polε, rescued both origin activation and cellular proliferation in *Pole4*^−/−^ cells*.*

Importantly, this model recapitulates the recently described genetic conditions caused by mutations of *MCM4*, *GINS1*, and *POLE1/POLE2* in humans (Pachlopnik Schmid et al., 2012; Logan et al., 2018; Frugoni et al., 2016; Cottineau et al., 2017; Hughes et al., 2012; Gineau et al., 2012). Notably, patients with hypomorphic mutation of *POLE*, in FILS and IMAGe syndromes, and *POLE2* exhibit a characteristic reduction in the number of B lymphocytes and variable degrees of immunodeficiency (Pachlopnik Schmid et al., 2012; Logan et al., 2018; Frugoni et al., 2016). By performing HU-EdU-seq, we observed that *Pole4*-deficient lymphocytes presented inefficient replication origin activation, independent of their genomic location, pointing to impaired origin activation as the primary pathogenetic mechanism at the basis of these immunodeficiencies. Unbiased high-resolution mapping of origin activation suggests that limiting levels of pre-IC components stochastically affects replication origin activation in this group of genetic diseases. We speculate that the specific expression levels of pre-IC components and the requirement for an elevated number of activated replication origins may explain the developmental and specific immunological defects associated with defective CMG activation in human patients.

In budding yeast, and during the early embryonic divisions in *Xenopus*, four initiation factors, SLD2/RECQL4, SLD3/TRESLIN, DBF4, and DPB11/TOPBP1, are limiting for origin activation and the control of S-phase length (Mantiero et al., 2011; Collart et al., 2017). While this remains to be clarified in mammalian organisms, our data show that Polε can act as the limiting factor for origin activation under specific experimental conditions.

We previously reported that loss of TRP53 rescues the phenotypical abnormalities observed in *Pole4*-deficient mice (Bellelli et al., 2018a). We show here that deletion of TRP53 rescues proliferation and reduces DNA damage accumulation in *Pole4*^−/−^ cells, via an unanticipated increase in Polε subunit levels. Cycloheximide and MG132 experiments showed that loss of POLE4 leads to proteasome-dependent degradation of Polε in the soluble but not the chromatin fraction of *Pole4*^−/−^ cells. Hence, once engaged with the CMG, it is likely that Polε remains sufficiently stable on replicating DNA, even in the absence of its POLE4 subunit. Loss of TRP53 in *Pole4*-deficient cells had no impact on the half-life of Polε, but instead sustained increased transcription levels of Polε subunits as revealed by RNA expression analysis. In particular, RNA-seq analysis in *Pole4*^+/+^ *Trp53*^−/−^ and *Pole4*^−/−^ *Trp53*^−/−^ cells supports a model where, under unchallenged conditions, loss of TRP53 dysregulates CDKN1A/P21 expression, leading to uncontrolled Cyclin-CDK activity, enhanced E2F-dependent transcription, and inappropriate origin activation. This phenomenon might be particularly important at the G1/S transition and in early S phase, where highly transcribed regions of the genome have to be replicated. Accordingly, work from several laboratories have established an important role for regulated CDKN1A/P21 degradation at S-phase onset (Abbas et al., 2008; Coleman et al., 2015).

P53 and P21 have been previously involved in several DNA metabolic transactions (for review see Sengupta and Harris, 2005; Cazzalini et al., 2010). Indeed, P53 can directly bind to RPA and RAD51 and suppress homologous recombination, while P21 can modulate translesional DNA synthesis by interacting with the polymerase processivity factor PCNA (Sengupta and Harris, 2005; Cazzalini et al., 2010). In addition to this, evidence has suggested a role for P53 and P21 in the control of replication origin activation or fork elongation in cancer cell lines. For instance, expression of P53 oncogenic variants has been recently reported to increase replication origin activation by controlling Cyclin A or CDC7 levels (Singh et al., 2017; Datta et al., 2017). Our findings in a genetic KO system clearly show that loss of TRP53 transcriptional activity itself promotes deregulated CDK activity and inappropriate origin activation. Our transcriptome analysis pointed to *Cdkn1A/p21* as the gene by far more downregulated upon genetic deletion of *Trp53*. In addition to this, the fact that transient or stable KO of CDKN1A/P21 recapitulates the altered replication dynamics of *Trp53*-deficient cells strongly suggests that loss of CDKN1A/P21 plays a major role in this phenomenon. While we cannot exclude that the functions reported for P53 and P21 in replication fork repair and restart might play additional roles in specific genomic contexts and/or in the presence of replication stressing agents or oncogenic stimuli, the rescue of replication fork rates by chemical inhibition of CDC7 suggests a major contribution of dysregulated origin activation in primary mammalian cells (Klusmann et al., 2016; Mansilla et al., 2016; Yeo et al., 2016; Maya-Mendoza et al., 2018; Roy et al., 2018). In line with this hypothesis, expression of a mutant of CDKN1A/P21 unable to inhibit CDKs (Chen et al., 1995; Cayrol et al., 1998) failed to restore replication fork rates as well as interorigin distance in primary *Cdkn1A/p21* KO cells. In contrast, expression of the PCNA binding mutant of P21 restored normal replication dynamics, similar to the WT counterpart (Chen et al., 1995; Abbas et al., 2008). Consistent with this, previous work had suggested that disruption of the PCNA binding domain of P21 has no major effect on the S phase of the cell cycle even in human cancer cells, in contrast to its N-terminal CDK inhibitory one (Nakanishi et al., 1995; Ogryzko et al., 1997).

Both *Trp53* and *Cdkn1A/p21* primary KO cells showed prominent signs of fork asymmetry, indicating the occurrence of transient and/or permanent fork stalling events. While we cannot completely exclude other possibilities, we speculate that increased origin activation and the consequent consumption of nucleotides and replication factors might represent the main mechanism to explain this phenomenon, in line with what was reported upon oncogene overexpression *in vitro* (Bester et al., 2011; Macheret and Halazonetis, 2015; Técher et al., 2017).

Finally, increased replication origin activation upon loss of TRP53-CDKN1A/P21 represents a therapeutic vulnerability for cancer cells. For instance, chemical ATR inhibition dysregulates origin activation and is particularly effective upon loss of P53 (Toledo et al., 2011). Accordingly, loss of TRP53 results in embryonic lethality in an ATR hypomorphic mouse model (Murga et al., 2009), which suggests that combined dysregulation of replication origin activation might be particularly toxic in this context.

In conclusion, our work provides a mechanistic explanation for a group of human genetic diseases affecting CMG activation and also establishes dysfunctional replication origin activation as a prominent mechanism inducing genome instability upon loss of the TRP53 and CDKN1A/P21 tumor suppressors.

### Limitations of the study

This work has been performed in primary murine cells grown under low oxygen conditions. While we took advantage of clean genetic KO systems, we cannot exclude that the expression of mutant forms of p53 in cancer cells might compromise DNA replication in additional manners.

Our HU-EdU-seq experiments show a strong reduction of origin activation in Polε hypomorphic cells. This technique detects origin activation in large replication initiation zones, in cell populations. Thus, it is possible that differential effects on initiation at individual origins are not significantly detected.

Furthermore, while our data showed a reduction in origin activation in Polε hypomorphic cells, exactly which step of origin activation is affected remains to be identified. Indeed, while studies in budding yeast have shown that loss of Polε compromises CMG formation (Bell and Labib, 2016), we cannot exclude that in mammalian cells origin activation is affected at a later stage (e.g., at the CMG activation step). In addition to this, while Polε levels are clearly limiting for replication origin activation in Polε hypomorphic cells, whether Polε acts as a limiting factor for origin activation in primary WT cells remains to be investigated.

Finally, by using CSK-Triton extraction methods, we observed destabilization of Polε in the soluble but not the chromatin fraction of *Pole4*^−/−^ cells. Since the soluble fraction is constituted by both cytoplasm and nucleoplasm, we cannot completely exclude that, in the absence of POLE4, Polε is degraded in the nucleoplasm upon unstable binding to chromatin.

## STAR★Methods

### Key resources table

**Table:** 

REAGENT or RESOURCE	SOURCE	IDENTIFIER
**Antibodies**		

Goat Anti-Rat IgG (H + L) Antibody, Alexa Fluor 594 Conjugated	Thermo Fisher	Cat#A-11007; RRID: AB_141374
Rabbit Anti-Mouse IgG (H + L) Antibody, Alexa Fluor488 Conjugated	Thermo Fisher	Cat#A-11059; RRID: AB_142495
Goat Anti-Rabbit IgG (H + L) Antibody, Alexa Fluor488 Conjugated	Thermo Fisher	Cat#A-11034
Peroxidase-conjugated Goat anti-Mouse IgG (H+L)	Thermo Fisher Scientific	Cat#G-21040; RRID: AB_2536527
Peroxidase-conjugated Goat anti-Rabbit IgG (H+L)	Thermo Fisher Scientific	Cat#G-21234; RRID: AB_2536530
Rabbit polyclonal anti-53BP1	Novus Biologicals	Cat#NB100-304; RRID: AB_10003037
Rabbit polyclonal anti-POLE4	This study	N/A
Mouse Monoclonal Anti-POLE2	Abcam	Cat#ab57298; RRID: AB_2166739
Rabbit polyclonal Anti POLE	Genetex	Cat#GTX132100
Rabbit polyclonal anti-POLD1	Bethyl	Cat#A304-007A; RRID: AB_2620355
Mouse monoclonal anti-MCM2	BD Biosciences	Cat#610701; RRID: AB_398024
Mouse monoclonal anti-Histone H1	Millipore	Cat#05-457
Rabbit polyclonal anti Mcm2 pS53	abcam	abcam Cat#ab109133
Mouse Monoclonal anti-PCNA	Santa Cruz Biotechnology	Cat# sc-56; RRID: AB_628110
Mouse Monoclonal anti-p21	BD Biosciences	Cat#556430
Mouse Monoclonal anti-Tubulin	Sigma-Aldrich	Cat#T6074; RRID: AB_477582
Rat monoclonal anti-BrdU	AbD Serotec	Cat#OBT0030
Mouse monoclonal anti-BrdU	Becton Dickinson	Cat#347580
Mouse Anti-Lamin A/C	Santa Cruz Biotechnology	Cat# sc-376248
Mouse Anti E2F1	Santa Cruz Biotechnology	Cat# sc-251

**Chemicals, peptides, and recombinant proteins**		

PhosSTOP phosphatase inhibitor cocktail	Roche	Cat#PHOSS-RO
EDTA-free Complete protease inhibitor cocktail	Roche	Cat#COEDTAF-RO
CldU	Sigma-Aldrich	Cat#C6891
EdU	Thermo Fisher Scientific	Cat#A10044
Biotin-Azide	Thermo Fisher Scientific	Cat#B10184
CuSO4	SIGMA	Cat#PHR1477
Sodium L-Ascorbate	SIGMA	Cat#A7631
Benzonase	Novagen	Cat#71206-3
DAPI	SIGMA	Cat#10236276001
Hydroxyurea	SIGMA	Cat#H8627
T4 Polynucleotide Kinase	(NEB)	Cat#M0201
T4 DNA polymerase (NEB)	NEB	Cat#M0203
Klenow fragment exo-	NEB	Cat#M0212
NEBNext dA-Tailing reaction buffer	(NEB)	Cat#B6059
KAPA HiFi HotStart ReadyMix (2X)	KAPA Biosystems	Cat# KK2600
MyOne Streptavidin C1 Beads	ThermoFisher	Cat #650-01
Quick Ligase	NEB	Cat #M2200L
Anti-CD43 (Ly-48) MicroBeads	Miltenyi Biotech	Cat# 130-049-80

**Critical commercial assays**		

Lipofectamine RNAiMAX	Thermo Fisher	Cat#13778150
QIAprep Spin Miniprep Kit	QIAGEN	Cat#27106
RNeasy Mini Kit	QIAGEN	Cat#74106
KAPA Library Quantification Kit	Kapa Biosystems	Cat# KK4824
QIA-quick Gel Extraction Kit	QIAGEN	Cat#28706
Click-iT EdU Alexa Fluor 647 Flow Cytometry Assay Kit	Invitrogen	Cat# C10424
Q5® Site-Directed Mutagenesis Kit	New England Biolabs	E0554S

**Deposited data**		

RNA-Seq dataset	This paper	GSE200475
HU-EdU-Seq datasets	This paper	GSE200331

**Experimental models: Cell lines**		

Mouse Embryonic Fibroblasts *Pole4*^*+/+*^*Trp53*^*+/+*^	This study	N/A
Mouse Embryonic Fibroblasts *Pole4*^*+/+*^*Trp53*^*+/-*^	This study	N/A
Mouse Embryonic Fibroblasts *Pole4*^*+/+*^*Trp53*^*-/-*^	This study	N/A
Mouse Embryonic Fibroblasts *Pole4*^*-/-*^*Trp53*^*+/+*^	This study	N/A
Mouse Embryonic Fibroblasts *Pole4*^*-/-*^*Trp53*^*+/-*^	This study	N/A
Mouse Embryonic Fibroblasts *Pole4*^*-/-*^*Trp53*^*-/-*^	This study	N/A
Mouse Embryonic Fibroblasts *Cdkn1a*^*+/+*^	Jackson Laboratory	Cat#016565
Mouse Embryonic Fibroblasts *Cdkn1a*^*-/-*^	Jackson Laboratory	Cat#016565

**Experimental models: Organisms/strains**		

*Pole4*^*tm1(KOMP)Vlcg*^	Bellelli et al. (2018a)	N/A
*Trp53*^*tm1Brd*^	Donehower et al. (1992)	N/A

**Oligonucleotides**		

ON-TARGETplus Non-targeting Control Pool	Dharmacon	Cat#D-001810-10
ON-TARGETplus Mouse Cdkn1a siRNA	Dharmacon	Cat#L-058636-00-0005
ON-TARGETplus Mouse Mdm2 siRNA	Dharmacon	Cat#L-041098-00-0005

**Software and algorithms**		

Adobe Photoshop CC	Adobe	http://www.adobe.com/es/products/photoshop.html
ImageJ	NIH	https://imagej.nih.gov/ij/
Volocity 6.3	PerkinElmer	http://cellularimaging.perkinelmer.com/downloads/detail.php?id=14
GraphPad Prism 7	GraphPad	https://www.graphpad.com/
R	R core team	https://www.r-project.org/
FlowJo (10.1)	FlowJo	https://www.flowjo.com/solutions/flowjo/
FastQC	Babraham Bioinformatics	https://www.bioinformatics.babraham.ac.uk/projects/fastqc/
DESeq2	Love et al. (2014)	http://www.bioconductor.org/packages/release/bioc/html/DESeq2.html
RSEM	Li and Dewey (2011)	http://deweylab.biostat.wisc.edu/rsem
Trim Galore!	Babraham Bioinformatics	https://www.bioinformatics.babraham.ac.uk/projects/trim_galore/

### Resource availability

#### Lead contact

Further information and requests for reagents should be directed to and will be fulfilled by the Lead Contact, Roberto Bellelli (r.bellelli@qmul.ac.uk).

#### Materials availability

Mouse cell lines generated in this study are available upon request to the Lead Contact, Roberto Bellelli (r.bellelli@qmul.ac.uk).

### Experimental model and subject details

#### Mouse strains and cell lines

Mouse strains and cell lines used in the study are listed in key resource table. Mouse Embryonic Fibroblasts were produced at embryonic day 13.5 from timed breeding between 8-12 weeks old *Pole4*^*+/−-*^ *Trp53*^*+/-*^ males and females mice in C57BL/6 background. All animal experimentations were undertaken in compliance with UK Home Office legislation (project license number 70/8527) under the Animals (Scientific Procedures) Act 1986. Primary *Pole4*^*+/+*^ and *Pole4*^*-/-*^ MEFs in a *Trp53*^*+/+ , +/-*^ *and* ^*-/-*^ background were cultured at 37°C/ 5% CO_2_/ 5% O_2_ in Dulbecco's modified Eagle's medium (DMEM) (Invitrogen) supplemented with 15% fetal bovine serum (FBS) (Sigma) and 1% penicillin-streptomycin (Invitrogen). Primary splenocytes from 6-12 week old *Pole4*^*+/+*^ and *Pole4*^*-/-*^ sex-matched male and female mice were purified as previously described (18). Resting B cells were isolated from wild-type and *Pole4* KO mouse spleens with anti-CD43 MicroBeads (Miltenyi Biotech) and cultured in RPMI with 10% FBS. *Cdkn1a*^+/+^ and ^-/-^ primary MEFs were kindly provided by Valery krizhanovsky, Weizmann Institute of Science, Israel.

### Method details

#### Mouse embryonic fibroblasts (MEFs) isolation and culture

*Pole4*^*+/-*^*Trp53*^*+/-*^ male and female mice in C57BL/6 background were mated. Pregnant females at 13.5 days gestation were subjected to euthanasia under anaesthesia, followed by uterine dissection to isolate individual embryos. Each embryo was washed in PBS followed by removal of head (used for genotyping) and internal organs (heart and liver). The embryo body was minced with sterile razor blades and incubated in trypsin at 37°C for 20 min, followed by gentle pipetting of the trypsin digest. Cell suspension was pelleted, resuspended and plated in 10 cm dishes (now considered passage 0) in DMEM (Dulbecco’s modified Eagle’s medium (DMEM) supplemented with 15% FBS (SIGMA) and 50μg/mL penicillin-streptomycin, 2mM L-glutamine. Once subconfluent, a standard 3T3 protocol was followed: every 3 days cells were trypsinized, counted using cellometer Auto 2000 (Nexcelom Bioscience) to determine the number of Population doublings (PD) and then replated at a fixed density (8x10^5^ cells per 100-mm dish). The accumulation of population doubling level (PDL) was calculated using the formula ΔPDL = log(nh/ni)/log2, where ni is the initial number of cells and nh is the cell number at each passage.

#### Nascent DNA sequencing (HU-EdU-seq)

Nascent DNA sequencing (HU-EdU-Seq) was essentially performed as described in Tubbs et al. (2018). Briefly, resting B cells from *Pole4*^*+/+*^ and ^-/-^ spleens were activated with LPS (25 mg/mL; Sigma), IL-4 (5 ng/mL; Sigma) and RP105 (0.5 mg/mL; Sigma) and incubated with 20 μM EdU for 28 h in the presence of 10 mM hydroxyurea (HU). Pelleted cells were fixed in 90% methanol for 15 min on ice, washed with PBS, permeabilized with 0.2% Triton X-100, for 10 min on ice, and processed for Click-IT biotin-labeling in 10 μM Biotin Azide (ThermoFisher), 200 μM CuSO4 (Sigma), and 10 mM sodium ascorbate (Sigma), for 2 h, at R.T. in the dark. DNA was then recovered using Phenol:Chloroform:Isoamyl Alcohol (25:24:1, v/v) (Invitrogen), according to manufacturer’s instructions and sheared to 150-200 bp fragments using the Covaris S220 sonicator. Biotin-EdU labeled DNA fragments were purified using MyOne Streptavidin C1 Beads. Beads were washed in 1x Binding and Wash Buffer (1xBWB) (10 mM Tris-HCl pH8.0, 1 mM EDTA, 1 M NaCl, 0.1% Tween 20) and recovered using a DynaMag-2 magnetic separator (12321D, Invitrogen). Washed beads were resuspended in 130 μL 2xBWB (10 mM Tris-HCl pH8.0, 2 mM EDTA, 2 M NaCl) combined with the 130 μL of sonicated DNA and incubated at 24°C for 30 min in a ThermoMixer C at 400 rpm. Bead bound biotinylated DNA was then washed in 1xBWB, EB buffer, T4 ligase reaction buffer (NEB) and finally resuspended in 50 μL of end-repair reaction mix (0.4 mM of dNTPs, 2.7 U of T4 DNA polymerase (NEB), 9 U of T4 Polynucleotide Kinase (NEB) and 1 U of Klenow fragment (NEB)) and incubated at 24°C for 30 min in a ThermoMixer C at 400 rpm. Separated beads were washed again with 1xBWB, EB buffer and NEBNext dA-Tailing reaction buffer (NEB) and finally resuspended in 50 μL of A-tailing reaction with NEBNext dA-Tailing reaction buffer (NEB) and 20 U of Klenow fragment exo- (NEB) and incubated at 37°C for 30 min in a ThermoMixer C at 400 rpm. The supernatant was removed using a magnetic separator, beads were washed with NEBuffer 2, resuspended in 115 μL of Ligation reaction with Quick Ligase buffer (NEB), 6,000 U of Quick Ligase (NEB) and 5 nM annealed adaptor (Truseq truncated adaptor) and incubated at 25°C for 30 min in a ThermoMixer C at 400 rpm. Ligation was stopped by adding 50 mM of EDTA, and beads were washed with 1xBWB and EB. PCR amplification was performed in 50 μL reaction with 10 mM primers 5′-CAAGCAGAAGACGGCATACGAGATXXXXXXGTGACTGGAGTTCAGACGTGTGCTCTTCCGATC∗T-3′ and 5′-AATGATACGGCGACCACCGAGATCTACACTCTTTCCCTACACGACGCTCTTCCGATC∗T-3′, and 2X Kapa HiFi HotStart Ready mix (Kapa Biosciences) where ∗ represents a phosphothioratebond and NNNNNN a Truseq index sequence. PCR reactions were cleaned with AMPure XP beads, and 200-500 bp fragments were isolated on 2% agarose gel. Libraries were purified using QIA-quick Gel Extraction Kit (QIAGEN) and concentration was determined with KAPA Library Quantification Kit for Illumina Platforms (Kapa Biosystems). Sequencing was finally performed on the Illumina NextSeq 550 (75bp single end reads).

#### Western blot analysis of mouse tissues and cells

Mouse testis were snap-frozen in liquid nitrogen and subsequently lysed in RIPA buffer (150 mM NaCl, 100 mM Tri pH 7.5, 1% NP-40, 0.1% SDS, 0.5% sodium deoxycolate) containing protease and phosphatase inhibitors (ROCHE) using the precellys 24 tissue disruptor (Berlin technologies). Similarly, primary fibroblasts from *Pole4*^*+/+*^ and *Pole4*^*-/-*^ embryos (MEFs) in a *Trp53*^*+/+*^, ^*+/-*^ or ^*-/-*^ background were lysed in RIPA containing protease and phosphatase inhibitors. Lysates were clarified by centrifugation (12.300 rpm 30 min at 4°C) and protein concentration was estimated by BRADFORD assay (SIGMA). Equal amounts of proteins were loaded on NuPAGE 4-12% Bis-Tris gels and transferred onto nitrocellulose membrane (Amersham). Membranes were blocked in 5% milk in PBST (PBS-Tween 0.1%) and incubated with primary antibodies and HRP-conjugated secondary antibodies.

#### Cycloheximide chase and treatment with MG-132

Primary MEFs were seeded in 6 cm dishes, grown to 70-80% confluency and then treated with 50 μg/mL cycloheximide (Sigma) in the presence or not of 20 μM MG-132 (Sigma) for the indicated time points. Cells were then lysed in RIPA buffer or chromatin was isolated using CSK-Triton extraction as described.

#### siRNA transfection

Passage 1 primary MEFs were grown to 40-50 % confluency and transfected with 20 μM siRNAs against mouse *Cdkn1a, Mdm2* or a negative control (ON TARGETplus SMARTpool, Dharmacon) using Lipofectamine RNAiMAX (Thermo Fisher) according to manufacturer instructions. siRNAs and Lipofectamine were initially diluted in Opti-MEM Medium and mixed after 5 min incubation. After an additional 15 min, the transfection mix was directly added to the cells. 48 h later, cells were lysed for Western blot or incubated with CldU and IdU for fiber stretching assay.

#### Chromatin fractionation

Chromatin fractionation experiments were performed as described in Bellelli et al., 2018a). Briefly, primary MEFs in mid-esponential phase of growth were washed once in ice-cold 1X phosphate-buffered saline (PBS) and lysed in ice-cold CSK (10 mM PIPES, pH 6.8, 100mM NaCl, 300 mM sucrose, 1mM MgCl2, 1 mM EGTA, 1mM DTT) buffer containing 0.5% Triton X-100 (Pierce Biotechnology) and protease and phosphatase inhibitors (ROCHE) for 10 min on ice. Chromatin-bound and un-bound proteins were separated by low speed centrifugation (3,000 rpm, 3 min at 4°C). The pellett (chromatin fraction) was washed once in CSK 0.5% Triton and resuspended in Laemmli buffer 1X. Total fraction was obtained by direct cell lysis in 1X Laemmli buffer. For each fraction, protein amounts deriving from comparable number of cells were analyzsed by SDS-PAGE and Western blotting.

#### Immunofluorescence staining

For indirect immunofluorescence staining, cells were seeded on coverslips and fixed in 4% paraformaldehyde. After permeabilization with 0.5% Triton X-100 (5 min on ice), coverslips were blocked in 1% BSA/PBS and incubated with anti-53BP1 (Novus Biologicals, NB100-304) primary antibody in 0.5% BSA/PBS for 1h at room temperature. Coverslips were then washed 3 times in PBS and incubated with Alexa Fluor 488 goat anti-rabbit antibodies (Invitrogen) for 45 min at room temperature. After DAPI counterstaining, coverslips were mounted in Glycerol/PBS (1:1) and observed with Axio Imager.M2 (ZEISS) using the Volocity 6.3 software.

#### Retroviral transduction of primary MEFs

Retroviral particles were generated by transient transfection of Phoenix ECO with: pBABE PURO and pBABE PURO CDKN1A/P21 WT, CDK- and PCNA- mutants; pBABE BLAST and pBABE E2F1 BLAST; pDEST-LTR-FLAG-HA and pDEST-LTR- FLAG-HA-POLE1. Sovranatants were harvested 48 and 72 h after transfection, spinned down, filtered and used to infect passage 1 primary MEFs in a 1:1 ratio with fresh media and in the presence of 8μg/mL polybrene. After 48 h infected cells were selected with puromycin (2 μg/mL) for 48 h or Blasticidin (5 μg/mL) for 4-5 days.

#### Generation of pBABE Cdkn1A/p21 mutants

pBABE PURO Cdkn1A/p21 was obtained by Addgene (cat#78783). Mutations in the PCNA and CDK interation domains (Cayrol et al., 1998; Abbas et al., 2008) were generated using the Q5® Site-Directed Mutagenesis Kit (New England Biolabs, E0554S) according to manufacturer’s instruction and verified by sequencing.

#### Cell cycle analysis

Cell cycle analysis was performed in passage 2 primary MEFs. Briefly, cells were harvested and fixed in 70% ice-cold methanol. After washing in PBS 1X, cells were resuspended in a buffer containing 0.1% NP40, 50 mg/mL propidium iodide (PI) and 100 mg/mL RNase A for at least 30 min in the dark. Data were collected with a BD LSR Fortessa and analysed using FlowJo V.10 software.

#### Fiber stretching assay

DNA fiber assay was essentially performed as described in Bellelli et al., 2018a). Briefly, MEFs of the indicated genotypes were pulse labelled with 20 μM CldU for 20 min and subsequently pulse labelled with 200 μM IdU for 20 min. Cells were trypsinized, washed in PBS, counted and resuspended at a concentration of 5 × 10^5^ in PBS. 2.5 μL of cell suspension were spotted on clean glass slides and lysed with 7.5 μL of 0.5% SDS in 200 mM Tris-HCL, pH 7.4, 50 mM EDTA (10 min, R.T.). Slides were tilted (15° to horizontal), allowing a stream of DNA to run slowly down the slide, air dried and then fixed in methanol/acetic acid (3:1) for 15 min at R.T. Acid-treated slides (45 min R.T.) were blocked in 1% BSA/PBS for 45 min at R.T. and incubated with rat anti-BrdU monoclonal antibody (1:1,000 over night; AbD Serotec) and mouse anti-BrdU monoclonal antibody (1:500 1h R.T.; Becton Dickinson). After 3 washes in PBS, slides were incubated with a mixture of Alexa Fluor 488 rabbit anti-mouse and Alexa Fluor 594 goat anti-rat antibodies (1:500 R.T.; Invitrogen) for 45 min at room temperature and mounted in PBS/Glycerol 1:1. Fibers were then examined using Axio Imager.M2 (ZEISS) with 60x oil immersion objective and the Volocity 6.3 software. For quantification at least 500 replication structures were counted per experiment.

#### RNA extraction and bulk RNA-Seq

RNA extraction from mouse embryonic fibroblasts was performed using the RNeasy Kit (Quiagen) according to manufacturer instructions. Sequencing was performed on a Illumina HiSeq 4,000 machine. The ‘Trim Galore!’ utility version 0.4.2 was used to remove sequencing adaptors and to quality trim individual reads with the q-parameter set to 20 (https://www.bioinformatics.babraham.ac.uk/projects/trim_galore/). Sequencing reads were then aligned to the mouse genome and transcriptome (Ensembl GRCm38 release-89) using RSEM version 1.3.0 (Li and Dewey, 2011) in conjunction with the STAR aligner version 2.5.2 (Dobin et al., 2012). Sequencing quality of individual samples was assessed using FASTQC version 0.11.5 (https://www.bioinformatics.babraham.ac.uk/projects/fastqc/) and RNA-SeQC version 1.1.8 (DeLuca et al., 2012). Differential gene expression was determined using the R-bioconductor package DESeq2 version 1.24.0 (Love et al., 2014; R Development Core Team, 2008). Gene set enrichment analysis (GSEA) was conducted as described in Subramanian et al. (2005).

### Quantification and statistical analysis

Statistics, including statistical tests used, number of events quantified, standard deviation, standard error of the mean, and statistical significance are reported in the figures and in the figure legends. Statistical analysis has been performed using GraphPad Prism7 software (GraphPad) and statistical significance is determined by the value of p < 0.05.

## Data Availability

• The accession numbers for the datasets reported in this paper are GSE200331 for the HU-EdU-Seq and GSE200475 for the RNA-Seq. • This paper does not report original code. • Any additional information required to reanalyze the data reported in this paper is available from the Lead Contact upon request.

## Supplemental information

Document S1. Figures S1–S6

Document S. Article plus supplemental informaion
