# Supplementary material for: Disrupted control of origin activation compromises genome integrity upon destabilization of Polε and dysfunction of the TRP53-CDKN1A/P21 axis
Source: Cell Rep. 2022 May 31;39(9):110871. doi: 10.1016/j.celrep.2022.110871 (PMC9637995; doi:10.1016/j.celrep.2022.110871)
Supplement: Document S1. Figures S1–S6 [file mmc1.pdf]

Cell Reports, Volume *39*

Supplemental information

Disrupted control of origin activation compromises genome integrity upon destabilization of Polε and dysfunction of the TRP53-CDKN1A/P21 axis

Valerie Borel, Stefan Boeing, Niek Van Wietmarschen, Sriram Sridharan, Bethany Rebekah Hill, Luigi Ombrato, Jimena Perez-Lloret, Deb Jackson, Robert Goldstone, Simon J. Boulton, Andre Nussenzweig, and Roberto Bellelli

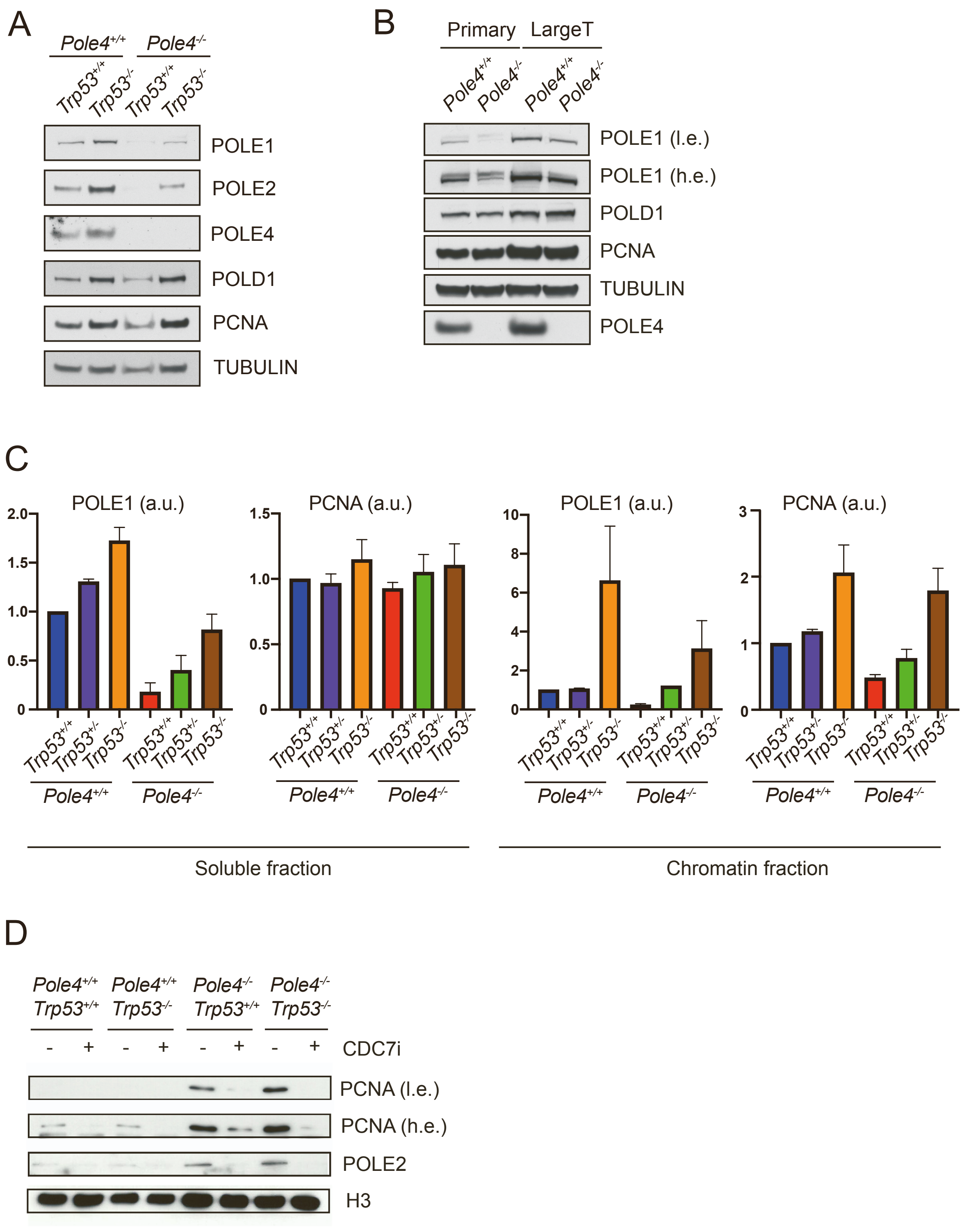

A
Pole4+/+
Pole4-/-
Trp53+/+
Trp53-/-
Trp53+/+
Trp53-/-
POLE1
POLE2
POLE4
POLD1
PCNA
TUBULIN
B
Primary
LargeT
Pole4+/+
Pole4-/-
Pole4+/+
Pole4-/-
POLE1 (l.e.)
POLE1 (h.e.)
POLD1
PCNA
TUBULIN
POLE4
C
POLE1 (a.u.)
PCNA (a.u.)
POLE1 (a.u.)
PCNA (a.u.)
Trp53+/+
Trp53+/-
Trp53-/-
Pole4+/+
Pole4-/-
Soluble fraction
Chromatin fraction
D
Pole4+/+ Trp53+/+
Pole4+/+ Trp53-/-
Pole4-/- Trp53+/+
Pole4-/- Trp53-/-
CDC7i
PCNA (l.e.)
PCNA (h.e.)
POLE2
H3

Figure S1

Figure S1 (related to Figure 2). Depletion of TRP53 rescues Polε subunits levels on total and chromatin fractions of *Pole4*$^{-/-}$ cells. (A) Western blot analysis of Polε subunits and replisome components from total extracts of *Pole4*$^{+/+}$ and $^{-/-}$ MEFs in a *Trp53* WT or KO background. Tubulin was used for normalization. (B) Western blot analysis of Polε subunits and replisome components from total extracts of *Pole4*$^{+/+}$ and $^{-/-}$ primary or Large T-immortalized MEFs. Tubulin was used for normalization. (C) Bar graphs showing relative intensity values of POLE1 and PCNA western blots from soluble and chromatin extracts of the described MEFs genotype. Results were normalized to *Pole4*$^{+/+}$ *Trp53*$^{+/+}$ MEFs and are reported as mean +/- SD of triplicate experiments. (D) Western blot analysis of POLE2 and PCNA levels from chromatin extracts of *Pole4*$^{+/+}$ and $^{-/-}$ MEFs in a *Trp53* WT or KO background treated or not with 5 μM CDC7i. Histone H3 was used for normalization.

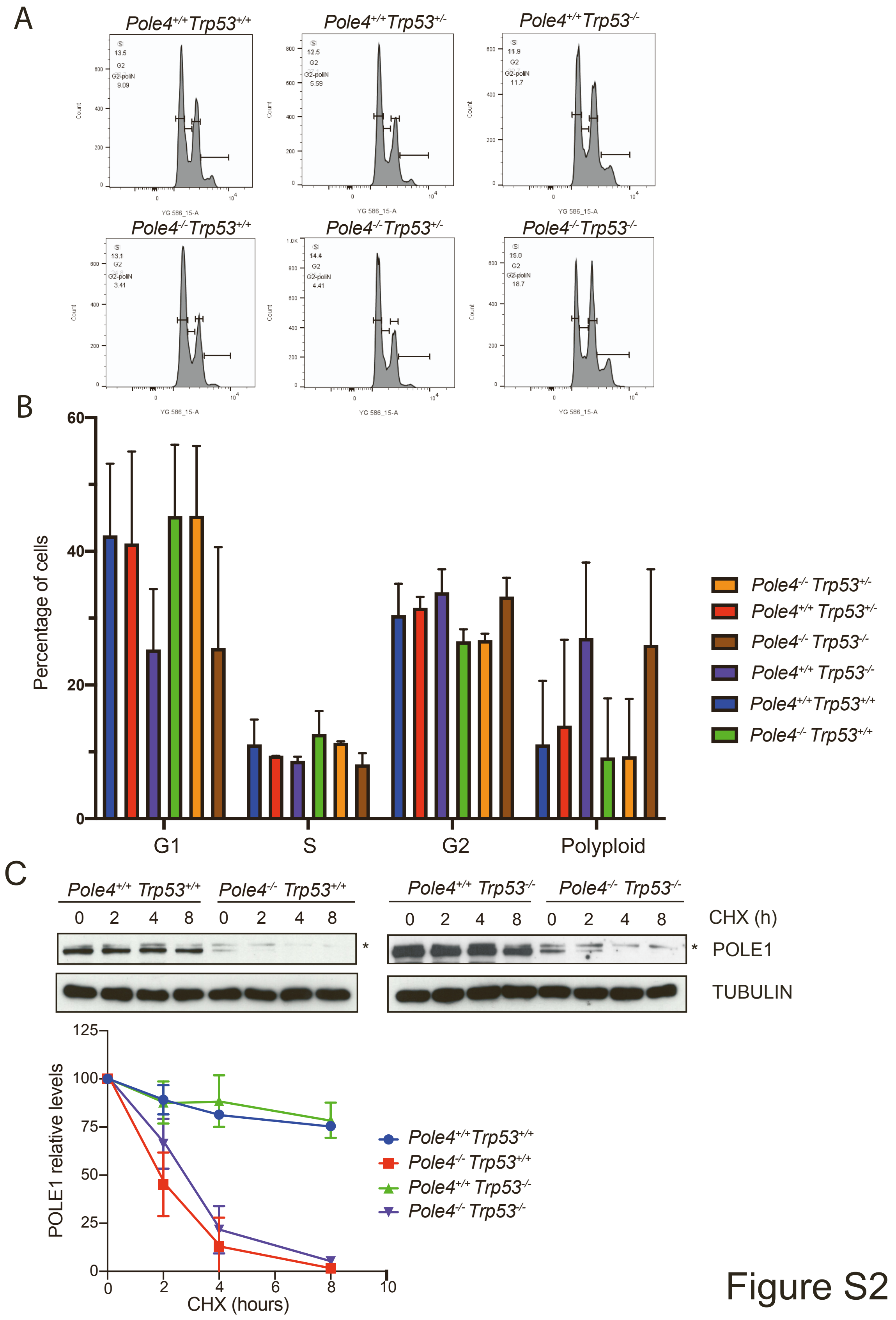
A
Pole4+/+ Trp53+/+
Pole4+/+ Trp53+/-
Pole4+/+ Trp53-/-
Pole4-/- Trp53+/+
Pole4-/- Trp53+/-
Pole4-/- Trp53-/-
Count
YG 586_15-A
B
Percentage of cells
G1
S
G2
Polyploid
Pole4-/- Trp53+/-
Pole4+/+ Trp53+/-
Pole4-/- Trp53-/-
Pole4+/+ Trp53-/-
Pole4+/+ Trp53+/+
Pole4-/- Trp53+/+
C
CHX (h)
POLE1
TUBULIN
POLE1 relative levels
CHX (hours)

Figure S2

Figure S2 (related to Figure 2). Deletion of *Trp53* rescues Polε subunits levels in a cell cycle-independent manner. (A) representative histogram plots showing propidium iodide incorporation (x axis) and cell count (y axis) for the indicated cell lines, analysed by flow cytometry. (B) Bar graphs showing the percentage of cells in G1, S, G2/M and polyploid (>4N) from the described genotypes. Results are reported as mean +/- SD of triplicate experiments. (C) Upper part: Western blot analysis of POLE1 levels in the soluble fraction of *Pole4*$^{+/+}$ and $^{-/-}$ MEFs in a *Trp53* WT (left panel) or KO (right panel) background, treated with CHX (Cycloheximide) for the indicated time points (h). Tubulin was used for normalization. The asterisk (*) in the POLE1 western blot indicates a non specific band. Lower part: half-life curve of POLE1 soluble levels in MEFs of the described genotypes, incubated with CHX for the indicated time points. Results are reported as mean +/- SD of triplicate experiments.

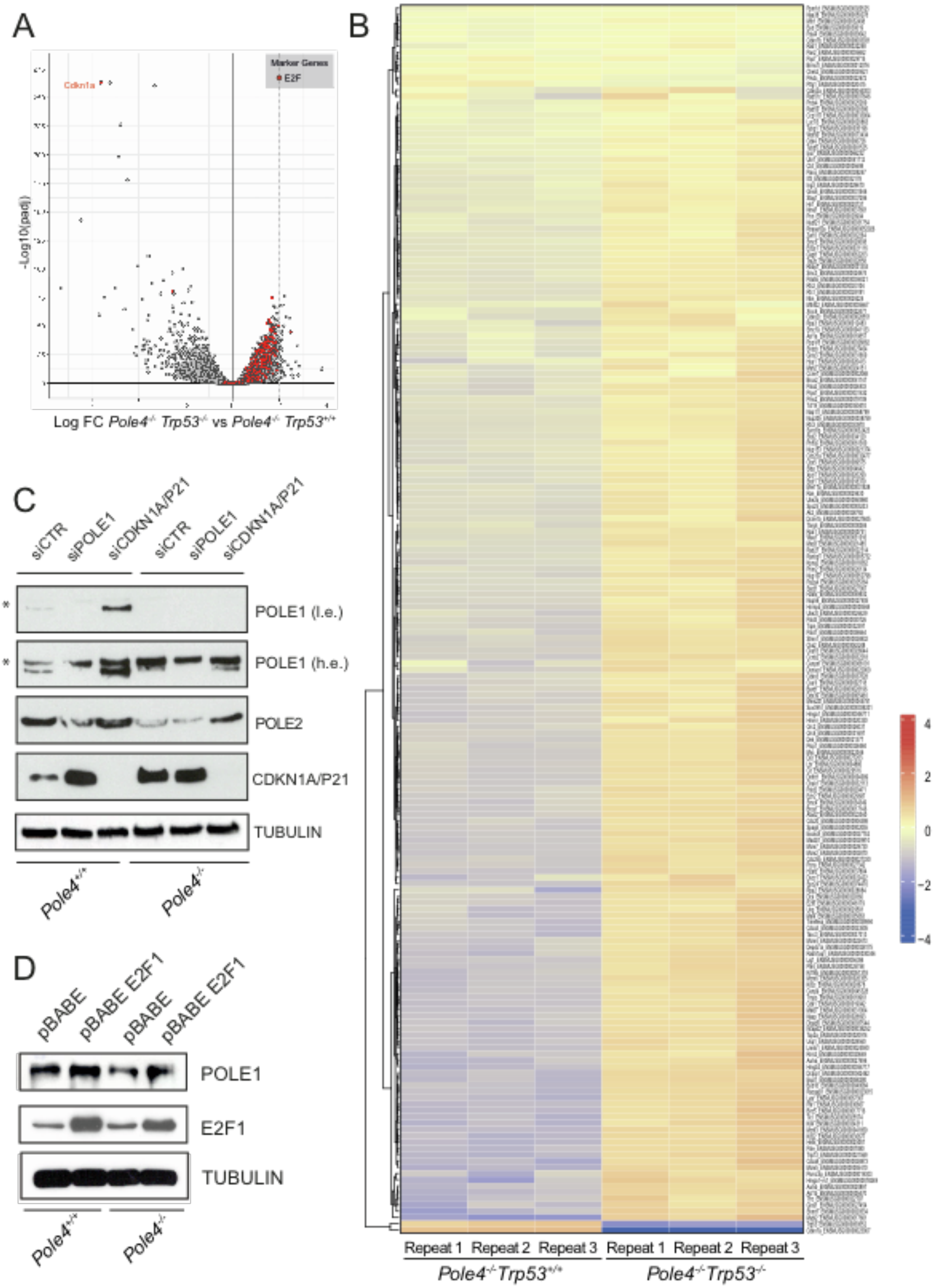
A
Cdkn1a
Marker Genes
E2F
-Log10(padj)
Log FC Pole4-/- Trp53-/- vs Pole4-/- Trp53+/+
B
Repeat 1
Repeat 2
Repeat 3
Repeat 1
Repeat 2
Repeat 3
Pole4-/- Trp53+/+
Pole4-/- Trp53-/-
4
2
0
-2
-4
C
siCTR
siPOLE1
siCDKN1A/P21
siCTR
siPOLE1
siCDKN1A/P21
POLE1 (l.e.)
POLE1 (h.e.)
POLE2
CDKN1A/P21
TUBULIN
Pole4+/+
Pole4-/-
D
pBABE
pBABE E2F1
pBABE
pBABE E2F1
POLE1
E2F1
TUBULIN
Pole4+/+
Pole4-/-

Figure S3

Figure S3 (related to Figure 3). Transcriptomic analysis of *Pole4*$^{-/-}$ cells in a *Trp53* WT and KO background reveals a E2F-dependent mechanism for increased Polε subunits levels. (A) Volcano Plot of RNA-Seq analysis displaying gene expression values for *Pole4*$^{+/+}$ *Trp53*$^{-/-}$ relative to *Pole4*$^{+/+}$ *Trp53*$^{+/+}$ MEFs. x axis represents the Log_2 fold change while the y axis represents the negative decade logarithm of the significance value change. Red dots indicate annotated *Trp53* downstream targets. *Cdkn1a/p21*, *Ccng1* and *Mdm2* are indicated. (B) Heatmaps of hallmark of E2F target genes from triplicate RNA-Seq of the indicated MEFs genotypes. (C) Western blot analysis of total cell extracts from *Pole4*$^{+/+}$ and $^{-/-}$ MEFs transfected with the indicated siRNAs. The asterisk (*) indicates a non specific band in the POLE1 western blot. Tubulin was used for normalization. (D) Western blot analysis of total cell extracts from *Pole4*$^{+/+}$ and $^{-/-}$ MEFs infected with retroviral vectors expressing E2F1 or a control. Tubulin was used for normalization.

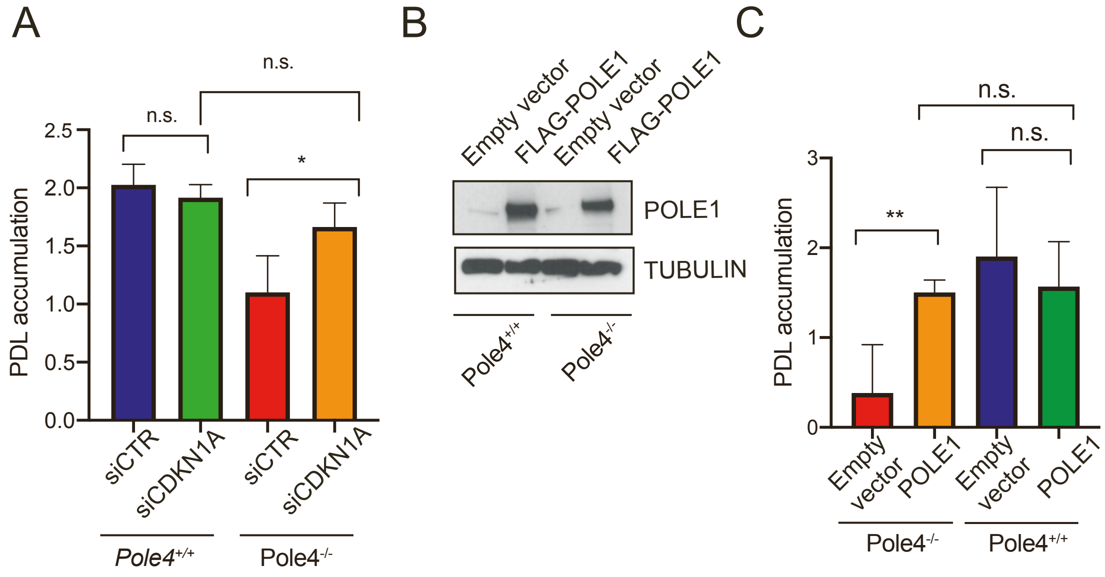

Figure S4

Figure S4 (related to Figure 4). Knock-down of CDKN1A/P21 and over-expression of POLE1 rescue growth rate in *Pole4* $^{-/-}$ cells (A) Bar graphs showing PDL (Population Doubling Level) accumulation of *Pole4*$^{+/+}$ and $^{-/-}$ MEFs transfected with siRNAs against *Cdkn1a/p21* or control siRNAs. Cells were transfected with siRNAs and, after 48 hours, seeded for population doubling counting. Results are reported as mean +/- SD of triplicate experiments; unpaired t- test analysis * p< 0.05, n.s. not significant. (B) Western blot analysis of total cell extracts from *Pole4*$^{+/+}$ and $^{-/-}$ MEFs infected with a retroviral vector expressing POLE1 or a control. Tubulin was used for normalization. (C) Bar graphs showing PDL (Population Doubling Level) accumulation of *Pole4*$^{+/+}$ and $^{-/-}$ MEFs infected with a retroviral vector expressing POLE1 or a control. Cells were infected with retroviral particles, selected with Blasticidin for 4-5 days and seeded for population doubling counting. unpaired t- test analysis ** p< 0.01, n.s. not significant. Results are reported as mean +/- SD of triplicate experiments.

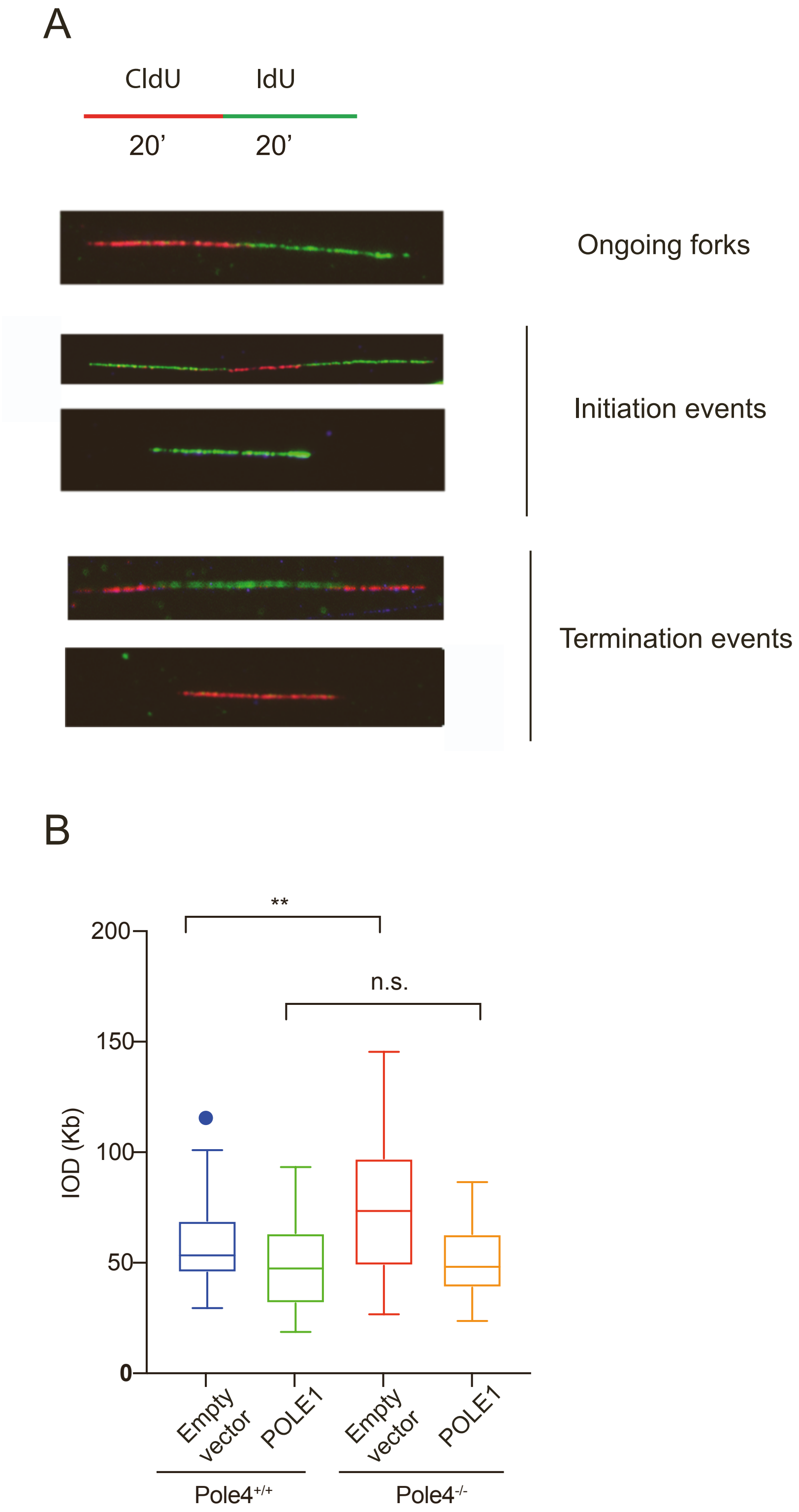
A
CldU
IdU
20'
20'
Ongoing forks
Initiation events
Termination events
B
**
n.s.
200
150
100
50
0
IOD (Kb)
Empty vector
POLE1
Empty vector
POLE1
Pole4+/+
Pole4-/-

Figure S5

Figure S5 (related to Figure 5). Replication structures observed upon labelling of cells with consecutive pulses of CldU and IdU and rescue of IOD upon over-expression of POLE1 in *Pole4* $^{-/-}$ cells. (A) Upper part: Scheme of the nucleotides pulse strategy used for the fiber stretching assay. Lower part: representative pictures of the replication fork structures analysed: ongoing replication forks, initiation events and termination events. (B) Bar graphs showing inter origin distance values from *Pole4* $^{+/+}$ and $^{-/-}$ MEFs infected with a retroviral vector expressing POLE1 or a control vector. unpaired t- test analysis ** $p< 0.01$, n.s. not significant. Fiber experiments have been performed four times and results are reported as box and whiskers plots using the tukey method.

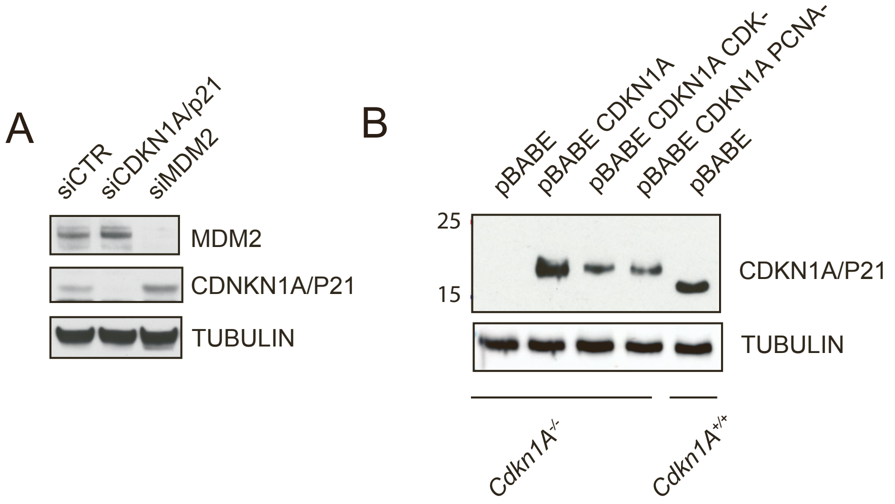

Figure S6

Figure S6 (related to Figure 7). Analysis of MDM2 and CDKN1A/p21 levels in primary cells transfected with the indicted siRNAs or expressing the indicated CDKN1A/p21 vectors. (A) Western blot analysis of total cell extracts from primary MEFs transfected with siRNAs against *Cdkn1a/p21*, *Mdm2* or control. Tubulin was used for normalization. (B) Western blot analysis of total cell extracts from primary *Cdkn1a/p21* $^{+/+}$ and $^{-/-}$ MEFs infected with the indicated retroviral vectors. Tubulin was used for normalization.
